# Bioprocess development as a sustainable platform for eco-friendly alkaline phosphatase production: an approach towards crab shells waste management

**DOI:** 10.1186/s12934-022-01868-4

**Published:** 2022-07-16

**Authors:** Soad A. Abdelgalil, Gaber A. Abo-Zaid

**Affiliations:** Bioprocess Development Department, Genetic Engineering and Biotechnology Research Institute (GEBRI), City of Scientific Research and Technological Applications (SRTA-City), Universities and Research Institutes Zone, New Borg El-Arab City, 21934 Alexandria Egypt

**Keywords:** Crab shell waste, Alkaline phosphatase, *Bacillus licheniformis*, Biovalorization, Mathematical experimental design, Spherical Central Composite, Batch fermentation, Eco-friendly production, Green remediation

## Abstract

**Background:**

There are substantial environmental and health risks associated with the seafood industry's waste of crab shells. In light of these facts, shellfish waste management is critical for environmental protection against hazardous waste produced from the processing industries. Undoubtedly, improved green production strategies, which are based on the notion of "Green Chemistry," are receiving a lot of attention. Therefore, this investigation shed light on green remediation of the potential hazardous crab shell waste for eco-friendly production of bacterial alkaline phosphatase (ALP) through bioprocessing development strategies.

**Results:**

It was discovered that by utilizing sequential statistical experimental designs, commencing with Plackett–Burman design and ending with spherical central composite design, and then followed by pH-uncontrolled cultivation conditions in a 7 L bench-top bioreactor, an innovative medium formulation could be developed that boosted ALP production from *Bacillus licheniformis* strain ALP3 to 212 U L^−1^. The highest yield of ALP was obtained after 22 h of incubation time with yield coefficient *Y*_*p/s*_ of 795 U g^−1^, which was 4.35-fold higher than those obtained in the shake-flask system. ALP activity has a substantial impact on the volatilization of crab shell particles, as shown by the results of several analytical techniques such as atomic absorption spectrometry, TGA, DSC, EDS, FTIR, and XRD.

**Conclusions:**

We highlighted in the current study that the biovalorization of crab shell waste and the production of cost-effective ALP were being combined and that this was accomplished via the use of a new and innovative medium formulation design for seafood waste management as well as scaling up production of ALP on the bench-top scale.

## Background

The environmental sustainability strategies based on molecular and complex natural products have the potential to become important stepping stones in the transition to a green circular economy in an era driven by global environmental change. The aquaculture industry is one of the most rapidly growing sub-sectors of the agricultural industry [1]. Seafood and other value-added products generated via processing are in high demand on the market. In recent years, the amount of marine garbage generated by processing industries has considerably grown. The generation of these marine by-products without the concurrent development of adequate recovery processes leads to environmental deterioration and serious pollution concerns. Seafood-based companies use roughly 25–30% of the total biomass for human consumption, while the other fractions are discarded as waste, with the shell making up the lion's share [2]. As a result, the aquaculture and seafood-processing sectors generate a significant amount of waste on a worldwide scale every year. Low biodegradation rates cause the majority of agricultural waste generated by the seafood processing plants to accumulate. Because of this, the removal of solid waste from the seafood industry has become a harrowing problem. It is very crucial to find a way to manage the waste that comes from processing seafood because it causes a lot of problems for the environment when it is thrown away in the ocean or on land [3]. This will, on the one hand, reduce the risk of diseases spreading due to air and water pollution caused by waste dumping, as well as prevent polluting the environment and fouling the earth. Furthermore, the recycling of seafood waste/bio-pollutants may, on the other side, provide industry-level production of vitally valuable materials/biomaterials [4]. Therefore, there is a great deal of interest in developing a plan for the efficient exploitation of industrial waste generated during the processing of seafood to reduce the environmental damage while also recovering economically valuable products. Crab shells are the seafood industry's most ubiquitous and significant trash. On a dry basis, the shells of the crustaceans have about 15–23% protein, 50–70% calcium (II), trioxocarbonate (IV) [CaCO_3_], and tricalcium diphosphate [Ca_3_(PO_4_)_2_], 15–30% chitin, and pigments (astaxanthin, canthaxanthin, lutein, or β-carotene). Depending on the crab and the season, these ratios might vary significantly [5]. For the exploitation of crab shells in various applications such as a source of chitin, metal removal from aqueous solutions, and drug delivery systems, several effective approaches have been proposed and are being used today [6]. Despite this, according to the authors' knowledge, no investigation has been conducted into the innovative and potentially effective utilization of carb shells as seafood waste in the production of functioning bacterial ALP to meet the needs of ever-increasing market demand. Alkaline phosphatases (APase, AP, or ALP; orthophosphoric monoester phosphohydrolase, EC 3.1.3.1) are metalloenzymes, non-specific monophosphoester hydrolase that catalyzes the hydrolysis of a broad range of phosphomonoesters as well as the transphosphorylation process by transferring the phosphoryl group to alcohol in the presence of appropriate phosphate acceptors at an alkaline pH condition [7]. Several organisms, ranging from prokaryotes to humans, have been shown to have ALPs, which are stable enzymes that remove phosphate groups from a wide variety of compounds. ALP plays a critical function in phosphate transport and metabolism as well as cell signalling, molecular activity control, and phosphate homeostasis in a wide variety of organisms. Moreover, compared to its human equivalent, the bacterial ALP has a substantially longer lifespan because of its intrinsic stability, therefore, they have garnered considerable attention in recent decades [8]. Environmental biotechnology, molecular biology, and immunodetection are just a few of the areas in which bacterial ALP has considerable potential as an experimental model for studying metal ion-dependent catalysis. Bacterial ALP throughput should be substantially enhanced to fulfil market and industry demand. This will be accomplished via the use of cost-effective and sustainable production strategies. Thus, bacterial ALP production in a cost-effective manner, by utilizing bioresources such as biowaste, as well as the effects of the whole bioprocessing process on the environment are two components of sustainable bioprocessing management [9].

**Fig. 1:**
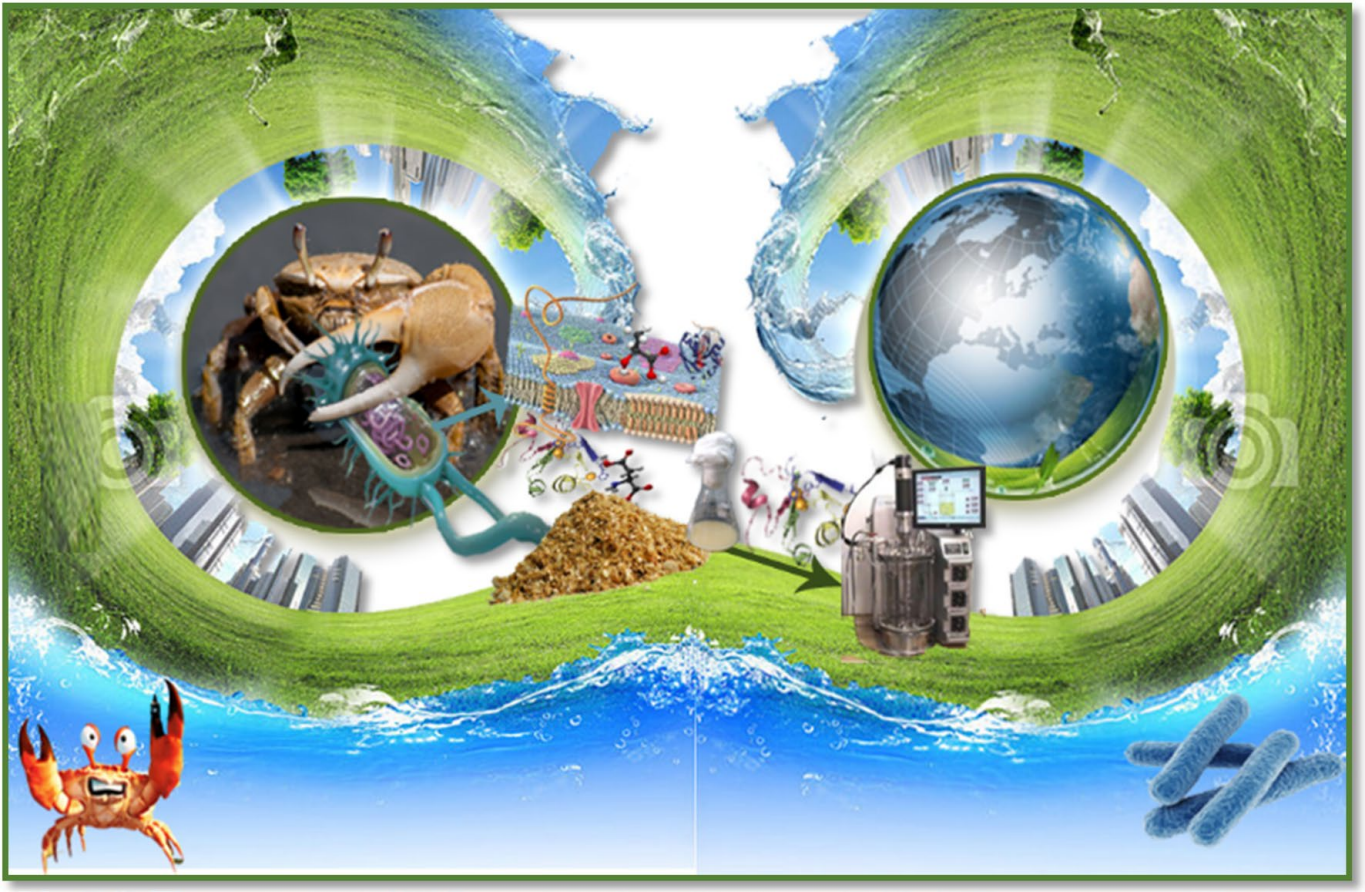
Schematic illustration of the exploitation of green chemistry sustainable strategies for the ultimate benefit of biowaste sugarcane molasses for the scale-up production of bacterial ALP on bench-top scale production

The optimization of process development should be seen as a vital aspect of the scaling-up process towards production. When it comes to media components, yield and efficacy of the process and other production characteristics, as well as plans for the next stage of the production process, this is the most important stage in the process. Process optimization based on models saves a significant amount of time and money compared to conducting experiments [10]. The statistical design of experiments is a strong method for statistical analysis and predictive modelling for systems for which our mechanistic understanding is insufficient, as is the case with many biological systems. Model introspection, along with statistical approaches, may lead to a better understanding of the mechanisms and processes [11]. It was necessary to rely on statistical experimental designs to create an innovative media formulation to produce ALP in the current research owing to a dearth of specialized literature on media formulation for microbial ALP production utilizing crab shells waste. However, due to the various culture modes, it is possible that the optimum parameters will not be immediately transferable from small batch to large fed-batch fermentations. Enlarging the bioreactor to a larger capacity while still keeping the outputs produced during laboratory or pilot scale studies is the goal of this approach [12]. A scale-up approach was also devised to contribute to the progress of knowledge and give technical direction for the commercialization process for industrial-scale production of ALP from *B. licheniformis* strain ALP3. As a result, seafood waste bioprocessing has the potential to generate substantial revenue via the exploitation of its by-products, in addition to being a step toward environmental protection and reduction of waste [13]. Therefore, the study's main goal was to produce bacterial ALP from crab shells using an ecofriendly approach to increase the utilization of crab shell trash and contribute to the reduction of environmental pollution. In this perspective, the current study was carried out to isolate a potent ALP-producing bacteria that was capable of degrading seafood waste efficiently and effectively. In addition, optimizations of metabolite production on a shake-flask scale followed by a scaling up of ALP production on a bench-top bioreactor scale were also addressed. Crab shell waste biovalorization and ALP production were combined for the first time in this paper, which employed a novel and unique medium formulation design to accomplish this goal as illustrated in Fig. 1.

## Results and discussion

### Isolation and identification of ALP-producing bacteria

**Fig. 2:**
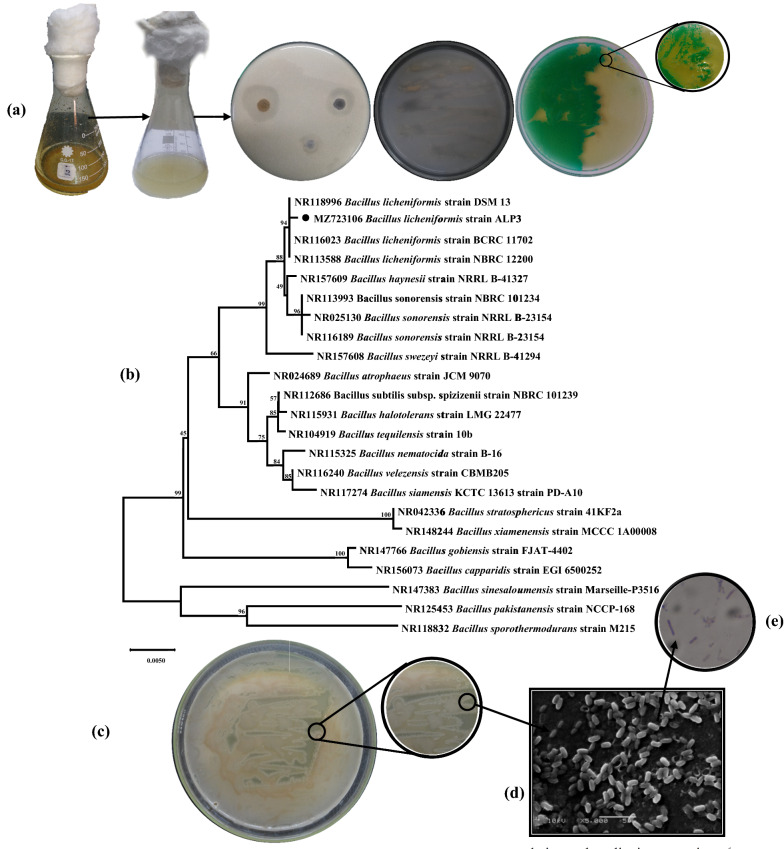
Isolation, screening, and identification of ALP-producing bacteria. **a** Isolation and qualitative screening of CaCO_3_-solubilizing and ALP-producing bacteria. **b** Phylogenetic tree based on *16S rDNA* gene sequence analysis showing the relationship of *B. licheniformis* strain ALP3 with reference strains (NCBI GenBank) constructed using the neighbor-joining method with the aid of MEGA X program. **c** Cultural feature of *B. licheniformis* strain ALP3 agar plate. **d** SEM micrograph of *B. licheniformis* strain ALP3 showing cell morphology at a magnification of 5000 and ×10,000  with 15 kv. **e** Gram-stain of the *B. licheniformis* strain ALP3 (using magnification, oil lens ×100)

ALPs have made considerable strides in both the scientific and bio-industrial fields. In light of the increasing need for ALP, it has become necessary to explore novel microbial isolates by researching diverse ecological niches to meet the growing demand [14, 15]. Because of this, sludge samples from the Alexandria tanning and leather factories were targeted in the bacterial isolation strategies program, and crab shells waste powder was added to the isolation broth medium in this investigation to boost the production of ALP, resulting in the isolation of 25 distinct morphotypes of isolates. It was based on the formation of halo zones on the PKV agar plates and colonies that were stained green when they broke down an artificial phenolphthalein diphosphate tetrasodium salt substrate that had been dyed with methyl green [7]. The ALP-producing bacteria with the ability to dissolve CaCO_3_ were identified and picked up. On PKV agar plates, the isolate labelled ALP3, which was chosen for further study, had the most visible colonies with a visible transparent zone. It also had the most bluish-green intense colonies in the PDP-methyl green agar plate and recorded the highest ALP activity of 48.3 U L^−1^ min^−1^ among the chosen isolates as illustrated in Fig. 2a. A nucleotide sequence length of 1355 bp was yielded from sequencing of the ALP3 isolate's *16S rRNA* gene, which was identified and submitted to a BLAST search in the GenBank database to compare and analyze with all those members of the genus *Bacillus*. GenBank has assigned the strain an accession number of MZ723106. As a consequence of the investigation, isolate ALP3 was shown to be genetically related to *B. licheniformis*, *B. haynesii*, and *B. sonorensis*, with sequence identities of 99.78, 99.56, and 99.41%. Besides that, a study found that the strain had 100% query coverage for all of its related species. It also had a total and maximum score of 2468, 2470, and 2459; respectively. An additional piece of evidence was presented in the form of neighbour-joining phylogenetic trees, which showed that this strain is part of the grouping of *Bacillus* and established different groups with other members of the group, as shown in Fig. 2b. Phylogenetic analysis of the *16S rRNA* gene sequences indicated that strain ALP3 grouped consistently with the genus *Bacillus* and created its distinct branch together with the type strain of *B. licheniformis* strain DSM 13, which had a bootstrap value of 94%. With the help of another cluster of *B. licheniformis* strain BCRC, this ALP3 phyletic line has been consistently recognized as belonging to the same clade as the *B. licheniformis* strain. Furthermore, the hereditary line of ALP3 formed a distinct clade with another cluster consisting of *B. haynesii* strain NRRL B-41327 and three additional *B. sonorensis* strains with a low bootstrap value (49%), which was different from the other cluster. This was in line with previous recent *B. licheniformis* genotyping investigations, which revealed that it is separated into at least two different groups [16]. Furthermore, *B. swezeyi* NRRL B-41294 established a clade with the ALP3 phyletic line that had an 88% bootstrap value. *B. licheniformis* strain ALP3 was proposed as a name for strain ALP3 based on taxonomic criteria since it strongly matches *B. licheniformis*. As part of the microscopical characterization of the strain ALP3, the research team found it to be gram-positive bacteria with rod-shaped cells of around 1.34 to 1.55 µm in length and 0.53 to 0.81 µm in width, as well as it is spore-forming bacteria (Fig. 2c). Cells are usually found alone, however, they may sometimes be found in chains of two cells that are motile and catalase-positive. Round and irregular-shaped colonies are found on the LB agar plate, with irregular (undulate and fimbriate) edges. It is common for colonies of *B. licheniformis* to have a rough, wrinkled surface covered with "licheniform," or hair-like growths. The colour fluctuates from opaque to white, and it may sometimes become crimson as illustrated in Fig. 2d. There have been a few investigations on the capacity of different bacteria to produce ALP. *E.coli*, *Thermotoga neapolitana*, *Thermus thermophiles*, *T*. *caldophilus*, *B. stearothermophilus*, and *B. licheniformis* (among others) are only a few of the kinds of bacteria that are reported as ALP-producing bacteria [15]. This work is unique in that it reports for the first time on the environmentally sustainable utilization of crab shells waste by bacteria in conjunction with the scaling up of ALP production from *B. licheniformis* in bench-top bioreactors based on the information available to the authors.

### Traditional optimization of the physical parameters for ALP production

**Fig. 3:**
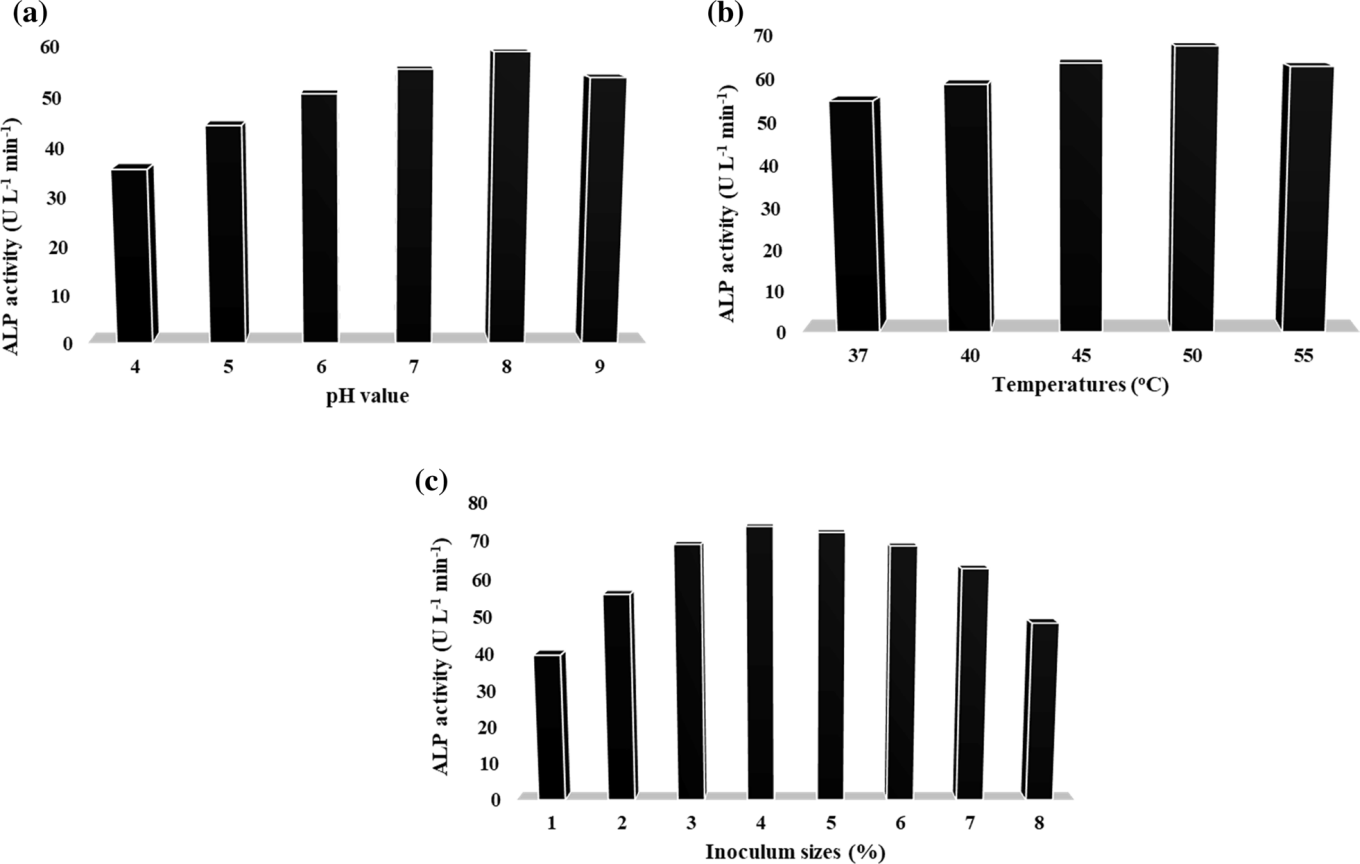
One Factor At Time optimization strategy of the physical parameters for ALP production from *B. licheniformis* strain ALP3. **a** Effect of different pH-values on the production of ALP; **b** Effect of different temperatures on the production of ALP; **c** Effect of different inoculum sizes on the production of ALP

It is necessary to identify the medium composition, pH, temperature, and other environmental factors that promote optimum growth and productivity [17]. The findings of this investigation indicate that the ALP production trend rises continuously from pH 4 (35.62 U L^−1^) to pH 8 (58.82 U L^−1^) before dipping back down to 53.8 U L^−1^ at pH 9 as shown in Fig. 3a. However, concerning maximal ALP production (63.87 U L^−1^), it seems that the unadjusted pH medium (pH 8.69–8.77) exhibits the best and most perfect conditions as compared to the adjusted pH medium with 1N HCl. As a result, *B. licheniformis* strain ALP3 has been shown to have a high predilection for alkaline environments when it comes to producing ALP. This conforms with earlier published results stating that alkaline conditions (pH 8.0 and 9.0) are needed for *B. paralicheniformis* APSO [7], *B. subtilis* [18], *B. licheniformis* [14], and *B. flexus* [19] to produce the highest amount of ALP. On the contrary, Abdelgalil et al. [20] and Dhaked et al. [21] reported that neutral pH (7.0) is the most favourable for high ALP production by *Lysinibacillus* strain APSO and *Bacillus* sp. Furthermore, the temperature of the environment has a significant impact on the functioning of cells. For every 10 °C rises in temperature, the growth rate almost doubles as the temperature approaches the ideal growth temperature [17]. The *B. licheniformis* strain ALP3 produced the most ALP (67.46 U L^−1^) when grown at 50 °C, according to the findings of this study. At temperatures lower than the optimal like 37 °C, transport of chemicals through cells is impeded, resulting in a less amount of enzymes being produced (54.88 U L^−1^) as illustrated in **Fig. ****3****b**. However, Behera et al. [22] and Abdelgalil et al. [7, 20] showed that the highest ALP production from *Alcaligenes faecalis*, *B. paralicheniformis* strain APSO, and *Lysinibacillus* strain APSO was achieved at 45 °C and then dropped subsequently, which is in contrast to the current findings. The inoculum concentration is a critical culture parameter for microbial growth and, therefore, industrial enzyme production. Due to quick substrate breakdown, there is a significant increase in industrial enzyme production by bacterial cells, which is associated with an increase in inoculum concentration until optimal inoculum size is obtained [17, 23]. According to the results of the present investigation, the 4% (v/v) of the activated pre-cultures was the best inoculum size for achieving maximum ALP production (73.82 U L^−1^). However, a low yield of ALP (48.37 U L^−1^) was noticed at higher inoculum levels (8%) due to the limitation of total dissolved oxygen and nutrition availability to bacteria as shown in Fig. 3c. According to earlier studies, the ideal inoculum size for the maximum ALP throughout was found to be 5% of the pre-activated culture of *B. paralicheniformis* strain APSO [6], *B. megaterium* [24]*,* and *B. subtilis* [18], which was aligned with the current findings. Other studies have shown that inoculum sizes of 1% are necessary for the production of ALPs by *E. coli* EFRL 13 [25] and *Lysinibacillus* strain APSO [20] in submerged fermentation, which contradicts the findings of this study.

### Statistical optimization of ALP production by *B. licheniformis* strain ALP3

Plackett–Burman's experimental design may be used to screen for medium variables that are critical to the experiment's performance. It is a partial factorial design in which many independent variables (N) are investigated in a relatively small number of experiments (N + 1) [26]. An accurate prediction of the main effects of independent factors on dependent variables is critical to the progression of ALP production. Plackett–Burman layout was used to create a structured experimental design matrix with twenty-six trials. This was done to screen the effects of thirteen different medium components, such as molasses, Arabic gum, NH_4_(SO_4_)_2_, NaNO_3_, crab shells waste powder, NaCl, MgCl_2_·6H_2_O, K_2_HPO_4_, NaH_2_PO_4_, CuSO_4_·5H_2_O, ZnSO_4_·7H_2_O, NiSO_4_, and CoCl_2_·6H_2_O corresponding to *X*_*1*_*–X*_*13*_, at their lowest and highest factor levels on ALP production. Table 1 shows the randomized PBD matrix that was used to screen the most critical elements impacting ALP production, as well as the average ALP activity of the trials and the anticipated ALP activity, which validates the model's appropriateness and exhibits a close match between experimental data and model values. The data listed in Table 1 demonstrate that the productivity of ALP varies substantially throughout the design matrix, ranging from 27.9 to 72.7 U L-1. This highlights the importance of medium optimization in boosting ALP efficiency. As shown by the fact that the maximum ALP activity (72.7 U L^−1^) was identified in trial number 3, which contained a high quantity of crab shells waste powder (*X*_*5*_; 25 g L^−1^) as indicated in Table 1. It is obvious from the obtained results that crab shells were necessary for the stimulation and production of ALP. However, trial number 5 showed that the ALP throughput was reduced to 27.9 U L^−1^ due to the usage of the smallest quantities of crab shells waste powder (2.5 g L^−1^). Consequently, the potential of crab shells in enhancing ALP production was highlighted and emphasized.

**Table 1:** Randomized Plackett–Burman experimental design for evaluating factors influencing ALP production by *B. licheniformis* strain ALP3

Trails	Variables													ALP productivity (U L^−1^ min^−1^)		
	X_1_	X_2_	X_3_	X_4_	X_5_	X_6_	X_7_	X_8_	X_9_	X_10_	X_11_	X_12_	X_13_	Actual value	Predicted value	Residual
1	1	–1	1	–1	–1	–1	–1	1	1	− 1	− 1	1	− 1	36.7881	35.95372	0.834416
2	–1	1	–1	1	–1	1	–1	–1	1	1	− 1	− 1	1	34.1707	38.6583	− 4.4875
3	–1	–1	–1	1	1	–1	–1	1	–1	− 1	1	1	1	72.7038	75.73725	− 3.03343
4	–1	1	–1	–1	–1	–1	1	1	–1	− 1	1	− 1	− 1	33.5891	37.43687	− 3.84771
5	1	–1	–1	1	–1	–1	1	1	1	1	− 1	1	− 1	27.9182	29.23588	− 1.31762
6	1	1	–1	1	–1	1	–1	–1	–1	− 1	1	1	− 1	36.3519	33.83077	2.521145
7	0	0	0	0	0	0	0	0	0	0	0	0	0	49.0023	48.19704	0.805335
8	0	0	0	0	0	0	0	0	0	0	0	0	0	49.0023	48.19704	0.805335
9	0	0	0	0	0	0	0	0	0	0	0	0	0	49.0023	48.19704	0.805335
10	–1	1	1	–1	–1	1	–1	–1	1	1	1	1	− 1	31.9896	33.56903	− 1.57935
11	1	1	1	1	1	1	1	1	1	1	1	1	1	47.2574	52.55927	− 5.30179
12	0	0	0	0	0	0	0	0	0	0	0	0	0	49.0023	48.19704	0.805335
13	0	0	0	0	0	0	0	0	0	0	0	0	0	49.0023	48.19704	0.805335
14	–1	1	–1	–1	1	1	1	1	–1	1	− 1	1	− 1	52.4921	50.55264	1.939514
15	–1	–1	–1	–1	1	1	–1	–1	1	− 1	− 1	1	1	65.4334	65.93677	− 0.50333
16	–1	–1	1	–1	–1	1	1	1	1	− 1	1	− 1	1	41.7319	41.45013	0.281867
17	0	0	0	0	0	0	0	0	0	0	0	0	0	49.0023	48.19704	0.805335
18	1	–1	1	–1	1	–1	–1	–1	− 1	1	1	− 1	− 1	61.6528	65.84953	− 4.19669
19	1	–1	–1	1	1	1	1	–1	1	− 1	1	− 1	− 1	58.7446	58.69547	0.049215
20	1	–1	–1	–1	–1	1	1	–1	–1	1	− 1	− 1	1	31.5534	32.34761	− 0.79415
21	1	1	1	–1	1	–1	1	–1	–1	− 1	− 1	1	1	52.3467	53.95518	− 1.60843
22	–1	–1	1	1	1	1	–1	1	–1	1	− 1	− 1	− 1	63.6885	63.0577	0.630845
23	1	1	1	1	–1	1	–1	1	–1	− 1	− 1	− 1	1	34.6070	34.26699	0.34003
24	1	1	–1	–1	1	–1	–1	1	1	1	1	− 1	1	70.8135	63.75566	7.057863
25	–1	–1	1	1	–1	–1	1	–1	–1	1	1	1	1	47.6937	42.06084	5.632868
26	–1	1	1	1	1	–1	1	1	1	− 1	− 1	− 1	− 1	57.5814	55.0312	2.550226

Variables	Code	− 1	0	+ 1
Molasses	X_1_	0.1	0.3	0.5
Arabic gum	X_2_	0.1	0.3	0.5
(NH_4_)_2_SO_4_	X_3_	0.02	0.11	0.2
NaNO_3_	X_4_	0.1	0.3	0.5
Crab shells powder	X_5_	2.5	13.75	25
NaCl	X_6_	0.1	0.3	0.5
MgCl_2_.6H_2_O	X_7_	0.02	0.06	0.1
K_2_HPO_4_	X_8_	0.02	0.06	0.1
NaH_2_PO_4_	X_9_	0.02	0.06	0.1
CuSO_4_.5H_2_O	X_10_	0.0005	0.0015	0.0025
ZnSO_4_.7H_2_O	X_11_	0.0005	0.0015	0.0025
NiSO_4_	X_12_	0.0005	0.0015	0.0025
CoCl_2_.6H_2_O	X_13_	0.0005	0.0015	0.0025

### Mathematical multiple regression analysis of PBD results

**Fig. 4:**
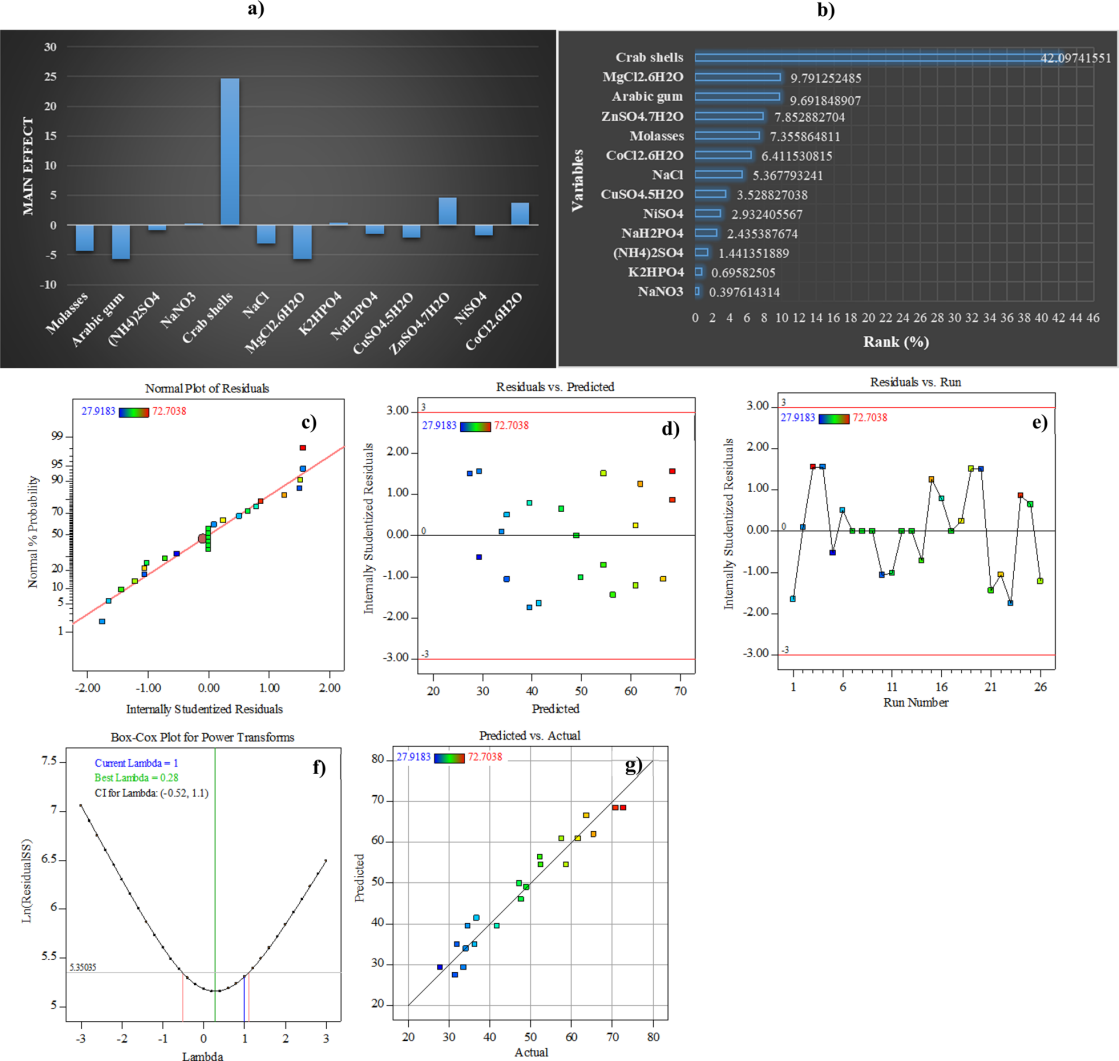
PBD results. **a** Main effect of culture variables. **b** Pareto chart illustrating the order and significance of the variables affecting ALP production by *B. licheniformis* strain ALP3. **c** Normal probability plot of the residuals for ALP production determined by the first-order polynomial equation. **d** Correlation between the residual and observation order. **e** Correlation between the residual and predicted values. **f** Box-Cox plot. **g** The plot of predicted versus actual ALP production

Table 2 shows the results of the regression analysis concerning the main effect, standard error, *t*-value, and *p*-value for each component. The main effects of all independent factors on ALP production are shown in Fig. 4a. ALP production was found to be significantly enhanced when crab shells waste powder (*X*_*5*_) was combined with ZnSO_4_·7H_2_O (*X*_*11*_) and CoCl_2_·6H_2_O (*X*_*13*_), according to the analysis of *t*-values. This suggests that the greatest concentration of these factors is recommended for the best ALP production. On other hand, ALP production was negatively affected by molasses, Arabic gum, (NH_4_)_2_SO_4_, NaCl, MgCl_2_.6H_2_O, NaH_2_PO_4_, CuSO_4_.5H_2_O, and NiSO_4_; nevertheless, NaNO_3_ and K_2_HPO_4_ had a minor effect although their presence in production medium at their zero level is needed to boost ALP productivity. According to the ANOVA results, which show that the contribution percentage of 42.097 of crab shells waste powder (*X*_*5*_) has a significant impact on the production process, as can be shown in Table 2, the *p*-value of 1.33E^−08^ and *t*-value of 13.46 also corroborate this finding. This study found that NaNO_3_ and K_2_HPO_4_ had high levels of confidence (97.26 and 93.71%, respectively) and also had a significant impact on the production of ALP with a *t*-value of 2.51, 2.05, respectively, the *p*-value of 0.0277, 0.0622 respectively, and contribution percentage of 7.85 and 6.41. The other coefficient variables that were included in this model had no discernible influence on the ALP yield.

**Table 2:** Statistical analysis of Plackett–Burman design showing coefficient values, *t*- and *p*-values for each variable affecting ALP production

Variables	Coefficient	Main effect	Std Error	*t*-Stat	*P*-value	*F*-ratio	Contribution %	Confidence level (%)
Intercept	48.197		0.80246	60.06	3E^−16^			100
*X*_*1*_	− 2.1520	− 4.30407	0.9149	− 2.352	0.0365	5.532	7.355	96.342
*X*_*2*_	− 2.8354	− 5.6709	0.9149	− 3.099	0.0092	9.603	9.691	99.079
*X*_*3*_	− 0.4216	− 0.84336	0.9149	− 0.4608	0.6531	0.212	1.441	34.687
*X*_*4*_	0.1163	0.232652	0.9149	0.1271	0.9009	0.016	0.3976	9.9063
*X*_*5*_	12.316	24.63205	0.9149	13.460	1.33E^−08^	181.3	42.097	100
*X*_*6*_	− 1.5704	− 3.14081	0.9149	− 1.7163	0.1117	2.945	5.367	88.822
*X*_*7*_	− 2.864	− 5.72906	0.9149	− 3.1307	0.0086	9.801	9.791	99.132
*X*_*8*_	0.2035	0.407141	0.9149	0.2224	0.8276	0.049	0.695	17.232
*X*_*9*_	− 0.712	− 1.42499	0.9149	− 0.7787	0.4512	0.606	2.435	54.877
*X*_*10*_	− 1.032	− 2.06479	0.9149	− 1.1283	0.2812	1.273	3.528	71.877
*X*_*11*_	2.297	4.594881	0.9149	2.5109	0.0273	6.305	7.852	97.264
*X*_*12*_	− 0.857	− 1.71581	0.9149	− 0.9376	0.3669	0.879	2.932	63.308
*X*_*13*_	1.8757	3.751517	0.9149	2.0501	0.0628	4.203	6.411	93.714

ANOVA	Df	SS	MS	*F*	Significance *F*		
Regression	13	3727.325	286.7173	17.1247	9.24E^−06^	Std. Dev	3.18
Residual	12	200.9142	16.74285			Mean	48.20
Total	25	3928.239				C.V.%	6.60
*R*^*2*^	0.948					PRESS	355.56
Adj. *R*^*2*^	0.93					Adeq. Precision	26.85
Pred. *R*^*2*^	0.909					Multiple *R*	0.974

The significant effects of the thirteen investigated independent variables on the process result were shown in decreasing order based on their *p*-values using a Pareto chart as an informative tool. A standardized Pareto chart's bars are proportionate to the absolute value of their related regression coefficients or estimated impacts [7]. According to Fig. 4b, crab shells waste powder was shown to be the most effective element among the variables tested, as evidenced by the Pareto chart, with a high degree of significance (*p*-value; 1.33E^−08^).

### Diagnosis of the statistical properties of the model

To evaluate the model's adaptability and establish whether or not there was a flaw in the experimental data, diagnostic plots were used. Normal plots may be used to examine the statistical characteristics of a model. The data points should be somewhat linear in form. Error term non-normality may be remedied by transforming the non-linear pattern (such as an S-shaped curve) [7, 27]. The ALP yield residuals have a normal distribution. There are no anomalies in the data based on their alignment with the drawn line [20]. As seen in Fig. 4c, the data points accumulated along the diagonal line demonstrating the adequacy of the model imply that the model is accurate [20]. In Fig. 4d, the residuals are plotted against the expected response, allowing for verification of the constant variance assumption. There was no way to predict which points in the experiments would be chosen by chance. Between 3 and -3, all of the values fell between these two numbers, which means that the models recommended by the PBD, as well as the assumption of constant variance were adequate [20]. As indicated in Fig. 4e, the residuals are plotted against the order in which the experimental runs were performed. It aims to determine if any unknown variables might have influenced the outcome of the experiment. Plots should be scattered randomly. The presence of trends suggests the presence of a time-related variable [7, 21]. The randomization process ensures that the analysis is not influenced by any trends that may emerge. Data on response may be transformed using the Box-Cox plot as a method of finding the most appropriate power transformation. The lowest point on the Box-Cox plot correlates to the λ value, which is the value in the modified model that produces the least amount of residual sum of squares. The power function may be used to explain the majority of data transformations; $\sigma =fn\left(\mu \alpha \right)$, where σ is the standard deviation and $\mu$ is the mean and the $\alpha$ is the power [27]. For a given observation, the transformation by λ power yields a scale that satisfies the statistical model's equal variance criteria, as the standard deviation is proportional to the mean raised to a power. The Box-Cox graph is better understood in Fig. 4f. The green line indicated the best λ-value 0.28) and λ transformation (λ = 1) was marked by the blue line, while the red lines symbolized the lowest and maximum values of confidence intervals between -0.52 and 1.1. There was no need for a recommendation for data transformation since the optimal value was found to be in the middle of the confidence intervals. In other words, the model was a good match for the experimental data [27]. When the predicted values were plotted against the actual data sets (as shown in Fig. 4g), it was discovered that the data sets were uniformly distributed along the 45° parallel. Although this may be used to identify cases in which the model is unable to smoothly anticipate a particular value or set of values; nonetheless, all data points are quantifiable, proving the model's appropriateness. The coefficient of determination (*R*^*2*^), on the other hand, may be used as an additional assurance of the fit's dependability in certain cases [28]. According to the findings in Table 2, the present model was demonstrated to explain more than 90% of the variation in response. The high value of *R*^*2*^ further highlighted that the model was well-fit, which added to the robust fit of the data set. In the light of the findings, the correlation coefficient (*R*^*2*^) was found to be 94.8% when the model was used. With the model accounting for just 5.2% of the variance that could not be explained by a linear model, it emerges that 94.8% of the experimental results were consistent with the model. For the regression model, a very high *F*-value (17.124) with low standard deviations value of 3.18 and a low probability (9.24E^−06^) means that the model is statistically significant at the 95% confidence level. A small value of standard deviations means that the model is well-fitting and that the predicted and real response values are close to each other. This shows us that it's a good model to use. Also, the high adjusted coefficient of determination (*R*^*2*^_adj_ = 0.93) as well as predicated *R*^*2*^; 0.909 shows that there is a close association between the model's significance and the variables that were studied, and the amount of ALPs that were made. When *R* (multiple correlation coefficient) approaches 1, the correlation between observed and anticipated values improves significantly. Here, *R* was shown to be a significant factor (0.974). The smaller the coefficient of variation (C.V.), the more accurate and reliable the experiments are. The current model exhibited a low coefficient of variation (CV = 6.60%) of an experiment's data which reflected the higher precision and reliability of the trials. On the other hand, acceptable accuracy suggests a signal-to-noise ratio should be greater than 4, which is desirable. An estimated signal-to-noise ratio of 26.85 indicates that there is sufficient signal to ensure that the model may be utilized to navigate across the design space. This model's residual error number of squares (PRESS) is determined to be 355.56 for the ALP productivity [29].

*Regression equation* The first-order model summarising the association between the thirteen parameters assessed across 26 trials and ALP activity, according to the ANOVA findings, is as follows:

$${Y}_{activity} = 48.19-2.15{X}_{1}-2.83{X}_{2}-0.421{X}_{3}+0.116{X}_{4}+12.31{X}_{5}-1.570{X}_{6}-2.864{X}_{7}+0.203{X}_{8}-0.712{X}_{9}-1.032{X}_{10}+2.297{X}_{11}-{0.857X}_{12}+1.875{X}_{13}$$

A verification experiment was used to corroborate the results of the Plackett–Burman design. Crab shells waste powder, 25; NaNO_3_, 0.3; K_2_HPO_4_, 0.06; ZnSO_4_·7H_2_O, 0.0025; CoCl_2_·6H_2_O, 0.0025, without pH adjustment and 4% activated inoculum size for 24 h incubation time were found to be the perfect conditions for the production of ALP at 50 °C and 200 rpm. This was a 1.22-fold increase above the activity observed before using the Plackett Burman design (73.82 U L^−1^) in all of these settings. As a result of this study's use of PBD approaches, an entirely new nutritive medium formulation was created, one that used simple, low-cost ingredients. Using statistical and experimental PBD, it is possible to improve bacterial ALP production, however, there are only a few publications on this. In contrast to the findings of previous researchers, Abdelgalil et al. [7] revealed that the most important factors that directly boosted ALP production by *B. paralicheniformis* strain APSO through PBD are molasses, (NH_4_)_2_SO_4_, and KCl; this finding was in opposition with the findings of this study. It also turns out that another study done by Abdelgalil et al. [20] stated that “molasses, NaNO_3_, and MgCl_2_·6H_2_O were the most important factors that made *Lysinibacillus* sp. APSO produces more ALP through PBD. This is in direct conflict with the findings of the present investigation. *Geobacillus thermodenitrificans* ALP production was improved by using PBD approaches that included glucose, yeast extract, and agitation as part of a multi-step process. However, NaCl, ZnSO_4_·7H_2_O, and CuSO_4_·5H_2_O all had a detrimental effect [30]; this finding contradicts that found in this research in terms of ZnSO_4_·7H_2_O while correlating with the current study's findings in terms of NaCl and CuSO_4_·5H_2_O. In addition, the most important factors boosting ALP production by *B. licheniformis* by PBD, according to Pandey et al. [14], were pH, temperature, fermentation duration, and orbital speed. There were additional published publications that relied on the one-factor-at-a-time (OFAT) approach to maximize bacterial ALP production. Sodium nitrate and molasses, among other factors studied by Qureshi et al. [25], had the largest influence on ALP from *E. coli* EFRL 13, which emphasizes the importance of NaNO_3_ in the ALP production process. Moreover, the other nutrients were studied to maximize the production of ALP, as reported that ALP production from *B. licheniformis* was shown to be boosted when the bacteria were grown in a medium containing glucose, peptone, and yeast extract, according to Pandey et al. [31], who discovered this while doing their study. Similarly, starch and casein were used to promote *Proteus mirabilis*'s ALP production. It was found that *Bacillus* spp. produced the most ALP when fed glucose and ammonium sulphate, according to Parhamfar et al. [32]. In addition, starch and egg albumin were determined by Jatoth et al. [18] to be suitable and function as boosters of *B. subtilis* ALP production.

In the last few years, there has been a lot of interest in using complex statistical optimization methods to improve ALP production and cut production costs. This is the third study of its kind that looks at this. This study followed this trend, and it looked at how to use food waste and low-cost nutrients to make extra bacterial ALP in the lab and cut down on the cost of the process through rigorous statistical optimization approaches.

### Response surface methodology (RSM)

RSM is an umbrella term for a set of statistical approaches that may be used to develop empirical models and evaluate them. It is often a critical concurrent engineering tool, since product design, process development, quality assurance, manufacturing engineering, and operations professionals frequently collaborate in a team setting to use RSM [33]. RSM designs aim to maximize the system's performance by optimizing a large number of factors concurrently. RSM was used to establish optimal conditions to find out the optimum levels of the most significant variables discovered by the PBD [34]. An embedded factorial or fractional factorial design with centre points typically referred to as a "central composite design," is strengthened with a number of "star points" that enable estimation of curvature. The central composite design has historically been considered to be one of the most essential designs for fitting second-order response surface models. As the experimental approach, the spherical CCD was chosen among other CCD kinds for this investigation. The CCD is spherical if α is set to a value of $\sqrt{k}$. All of the design points for spherical CCDs are located on the same geometric sphere in these systems. While spherical CCDs are not quite rotatable, they are pretty darn close [29]. Using an empirical half-factor spherical central composite (SCCD) model, the RSM model evaluated the interactions and modelling process of the dimensionless coded variables studied in the previous Plackett–Burman screening design (crab shells waste powder [X_5_], ZnSO_4_·7H_2_O [X_11_], and CoCl_2_·6H_2_O [X_13_]) and had a confidence level of 100, 97.26, and 93.71%, respectively. The other variable, on the other hand, had a negative influence on the ALP manufacturing process, according to the PBD findings; hence, these insignificant factors were left out from further optimization processes to enhance ALP production and cut production costs. As for the variables that had a small effect like NaNO_3_ and K_2_HPO_4_, their presence in the production medium at zero is needed to make ALP more productive. The SCCD conducted forty experiments with a highly precise random matrix of twelve axial points, sixteen factorial points, and twelve central points, as stated in Table 3, variable codes and values and the design matrix, as well as experimental and anticipated findings, were addressed for the variables selected. As seen in the following equation, the respective factors are coded as follows [29]: ${X}_{coded}=\frac{\left[{x}_{act}-{x}_{cen}\right]}{\left[\frac{{x}_{cen}-{x}_{min}}{\sqrt{n}}\right]}$

**Table 3:** Matrix designed for *B. licheniformis* strain ALP3 SCCD

StdOrder	Trials		Variables			ALP productivity (U L^−1^ min^−1^)		
		Type	X_1_	X_2_	X_3_	Actual value	Predicted value	Residual
27	1	Axial	0	0	1.73	117.78	121.806	− 4.0267
9	2	Factorial	− 1	− 1	1	100.62	99.4245	1.1975
12	3	Factorial	1	− 1	1	123.88	121.488	2.3985
15	4	Factorial	1	1	1	126.21	128.518	− 2.3046
34	5	Center	0	0	0	121.85	121.463	0.3877
21	6	Axial	0	− 1.73	0	113.12	113.826	− 0.6992
11	7	Factorial	1	− 1	1	122.72	121.488	1.2353
24	8	Axial	0	1.73	0	107.89	107.491	0.4014
30	9	Center	0	0	0	120.68	121.463	− 0.7755
37	10	Center	0	0	0	121.85	121.463	0.3877
14	11	Factorial	− 1	1	1	92.479	88.1329	4.3463
25	12	Axial	0	0	− 1.73	106.72	104.454	2.2748
16	13	Factorial	1	1	1	127.66	128.518	− 0.8505
33	14	Center	0	0	0	121.85	121.463	0.3877
2	15	Factorial	− 1	− 1	− 1	130.57	126.921	3.6548
18	16	Axial	− 1.73	0	0	88.989	93.4818	− 4.4923
4	17	Factorial	1	− 1	− 1	72.994	77.0086	− 4.0139
38	18	Center	0	0	0	121.85	121.463	0.3877
7	19 2	Factorial	1	1	− 1	79.683	80.9847	− 1.3013
20	20	Axial	1.73	0	0	87.535	85.2310	2.3043
36	21	Center	0	0	0	120.68	121.463	− 0.7755
40	22	Center	0	0	0	121.85	121.463	0.3877
29	23	Center	0	0	0	121.27	121.463	− 0.1938
31	24	Center	0	0	0	120.68	121.463	− 0.7755
6	25	Factorial	− 1	1	− 1	111.96	112.576	− 0.6121
22	26	Axial	0	− 1.73	0	112.54	113.826	− 1.2808
39	27	Center	0	0	0	121.85	121.463	0.3877
17	28	Axial	− 1.73	0	0	90.152	93.4818	− 3.3290
23	29	Axial	0	1.73	0	107.02	107.491	− 0.4709
19	30	Axial	1.73	0	0	88.698	85.2310	3.4675
5	31	Factorial	− 1	1	− 1	111.09	112.576	− 1.4846
28	32	Axial	0	0	1.73	119.23	121.806	− 2.5726
1	33	Factorial	− 1	− 1	− 1	127.95	126.921	1.0374
32	34	Center	0	0	0	121.27	121.463	− 0.1938
8	35	Factorial	1	1	− 1	80.265	80.9847	− 0.7197
3	36	Factorial	1	− 1	− 1	73.867	77.0086	− 3.1415
26	37	Axial	0	0	− 1.73	106.72	104.454	2.2748
35	38	Center	0	0	0	121.85	121.463	0.3877
10	39	Factorial	− 1	− 1	1	101.78	99.4245	2.3607
13	40	Factorial	− 1	1	1	92.479	88.1329	4.3463

Variables	Code	Coded and actual levels				
		− 1.73	− 1	0	1	1.73
Crab shells powder	X_1_	12	24	36	48	60
ZnSO_4_.7H_2_O	X_2_	0.001	0.002	0.003	0.004	0.005
CoCl_2_.6H_2_O	X_3_	0.001	0.002	0.003	0.004	0.005

Based on the three variables listed above, ALP activity was found to vary substantially in terms of efficiency. These results show a range of ALP activity of 72.99 to 130.57 U L^−1^. As demonstrated in Table 3, 130.57 and 127.95 U L^−1^ (predicted to be 126.92 U L^−1^) of ALP production were obtained at the factorial point when all chosen variables were at − 1 level, which is represented in experimental trials (number 15 and 33). On the other hand, when all predictors were maintained at − 1 levels, with the exception of crab shells waste powder, which was retained at its + 1 level, the experimental trials numbers 17 and 36 showed 72.99 and 73.86 U L^−1^ of ALP production, respectively (predicted to be 77 U L^−1^).

### Multiple regression analysis and ANOVA

Data from the SCCD experiment are summarised in Table 4, which shows that multiple regression and ANOVA calculations were used to assess the efficacy and applicability of the quadratic regression model. Additionally, linear, quadratic, and interaction effects as well as *p*-values at < (0.05) confidence levels were evaluated using ANOVA. The model's accuracy was also checked using *R*^*2*^, adjusted *R*^*2*^, predicted *R*^*2*^, and the sum of squares of prediction error (PRESS). The determination coefficient (*R*^*2*^) expresses the degree to which the regression equation fits the sample data effectively. While *R*^*2*^ may be increased by increasing the number of predictor variables in the model, it has been recommended to use an adjusted *R*^*2*^. An *R*^*2*^ that accounts for the number of explanatory variables in a model is known as an adjusted *R*^*2*^. A predicted *R*^*2*^ is used to figure out how much of the new data's variation the model can account for [28]. This study's ANOVA indicated an *R*^*2*^ determination coefficient of 0.981, showing that independent factors impacted ALP production by 98.1%; just 1.9% of the total difference driven by factors could not be described by this model and couldn't explain how ALPs worked. Even more critically, according to Koocheki et al. [35], only a high adjusted *R*^*2*^ can be utilized to establish this claim about a regression model's strength, even if the *R*^*2*^ is high. The results of the ANOVA revealed that the adjusted determination coefficient (*R*^*2*^_adj_) was 0.976, indicating that the model was statistically highly significant. It was found that the predicted *R*^*2*^ of 0.960 would be in accord with the adjusted *R*^*2*^, i.e. their discrepancy was less than the recommended value of 0.2. The model's multiple correlation coefficient (*R*) and standard deviation were 0.99 and 2.52, respectively. Good models have minimal standard deviations, which imply that the responses predicted by the model are close to those that occurred. The quadratic model was shown to be significant by ANOVA analysis with a *p*-value of 1.3276E^−23^ and an *F*-value of 180.17 as well as the low coefficient of variation (CV = 2.30%) of an experiment's data. Adequate precision is a measure of the signal-to-noise ratio, and it provides a factor by which you can analyze your model to determine whether it is appropriate to navigate through the design space and predict the response. Sufficient accuracy suggests a ratio greater than 4 in terms of signal-to-noise ratio [34]. According to the model, a signal-to-noise ratio of 40 indicates that the model may be utilized to traverse the design space. The predicted residual error sum of squares (PRESS) is a way to figure out how well a model from this experiment will likely predict the response in a new experiment. Predictability is better when the PRESS value is smaller, the ANOVA demonstrated that the model has a PRESS value of 410. The probability values (*p*-values) were computed to determine the significance of each coefficient, which is necessary for determining the independent factor's relevance [29]. It is also important to note that, according to the findings reported in Table 4, ALP production is boosted by the positive coefficients for the variables' linear effect *X*_*3*_, as well as by the positive coefficients for the variables' mutual effect *X*_*1*_*X*_*2*_, *X*_*1*_*X*_*3*_, and *X*_*2*_*X*_*3*_, which is shown by their *F*-values, *p*-values, *t*-values, and confidence levels, as well as their contribution%. The linear effects (*X*_*1*_ and *X*_*2*_) as well as the quadratic effects (*X*_*1*_^*2*^, *X*_*2*_^*2*^, and *X*_*3*_^*2*^) of the variables under examination, on the other hand, were shown to have an antagonistic effect. The negative sign of their coefficients and the computational suggestions for their significance degrees highlighted the fact that they had made no significant contribution to increasing ALP production by *B. licheniformis* strain ALP3. Nontrivial processes may benefit from the flexibility and precision provided by the second-order polynomial model, which is well-suited for both optimization and inference. *B. licheniformis* strain ALP3' alkaline phosphatase production (*Y*) might be explained by the second-order polynomial equation shown below, which was discovered using multiple regression analysis of the application data.

**Table 4:** Analysis of variance for the response surface of ALP production by *B. licheniformis* strain ALP3 obtained by SCCD

Term	Coefficient estimate	Mean square	*t*-Stat	*F*-value	*p*-value	Confidence level (%)	Contribution (%)
Intercept	121.46	1144.29	166.96	180.17	1.3276E^−23^		
*X*_*1*_	− 2.38	158.84	− 5.001	25.01	2.32312E^−05^	99.997	4.798133
*X*_*2*_	− 1.83	93.65	− 3.840	14.75	0.0006	99.940	3.68429
*X*_*3*_	5.01	702.59	10.51	110.63	1.39353E^−11^	100	10.0912
*X*_1_*X_*2*_	4.58	335.67	7.269	52.85	4.28744E^−08^	100	9.22715
*X*_1_*X_*3*_	17.99	5180.66	28.56	815.71	2.60377E^−23^	100	36.2495
*X*_2_*X_*3*_	0.7634	9.32	1.211	1.47	0.2351	76.490	1.53786
*X*_1_*X_*1*_	− 10.70	3608.10	− 23.83	568.11	4.73558E^−21^	100	21.5602
*X*_2_*X_*2*_	− 3.60	408.63	− 8.021	64.34	5.93074E^−09^	100	7.2557
*X*_3_*X_*3*_	− 2.78	243.05	− 6.186	38.27	8.29518E^−07^	96.999	5.5958

	df	SS	MS	*F-*value	Significance *F*		
Regression	9	10,298	1144	180.17	1.3276E^−23^	Std. Dev	2.52
Residual	30	190.53	6.351			Mean	109.51
Total	39	10,489				C.V. %	2.30
Multiple *R*	0.99					PRESS	410.68
R^2^	0.981					Adeq. Precision	40.87
Adj. *R*^*2*^	0.976						
Pred. *R*^*2*^	0.960						

“Std. Dev. is the standard deviation, the coefficient of determination (*R*^*2*^), Adj *R*^*2*^ is the adjusted-*R*^*2*^, and PRESS is the prediction error sum of squares, C.V is the coefficient of variation

$${Y}_{activity }=121.46-2.38{X}_{1}-1.83{X}_{2}+5.01{X}_{3}+4.58{X}_{1}{X}_{2}+17.99{X}_{1}{X}_{3}+0.763{X}_{2}{X}_{3}-10.70{X}_{1}^{2}-3.60{X}_{2}^{2}-2.78{X}_{3}^{2}$$

where *Y* is the predicted response (ALP activity), and *X*_*1*_, *X*_*2*_, and *X*_*3*_ are the coded levels of the independent variables of crab shells waste powder, ZnSO_4_.7H_2_O, and CoCl_2_.6H_2_O, respectively.

### Model adequacy checking

**Fig. 5:**
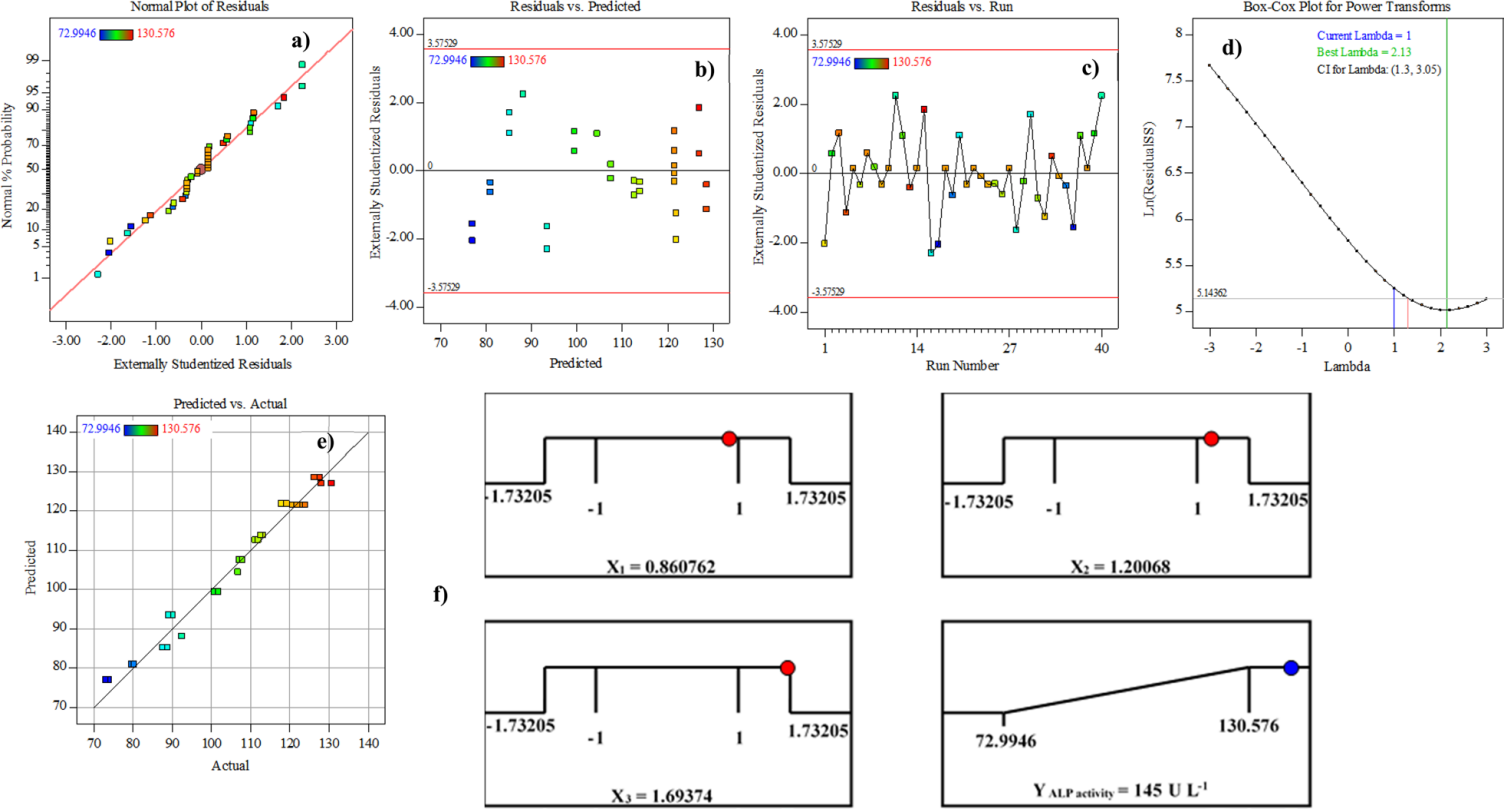
Model adequacy checking of Spherical-CCD: **a** Normal probability plot of the residuals. **b** Externally studentized residuals versus predicted ALP production. **c** Correlation between the residual and observation order. **d** Box-Cox plot. **e** The plot of predicted versus actual ALP production. **f** The optimization plot displays the desirability function and the optimum predicted values for the maximum ALP production

The model's adequacy was assessed using diagnostic plots, which helped to identify any issues in the experimental data and to provide a definitive solution [27]. Checking the model's adequacy necessitates the use of the normal probability plot. Because the data points are tightly clustered along the straight line, it means that the residuals have a normal distribution with linearity, this is shown in Fig. 5a. These values were found to correlate quite well, and values that varied significantly from the overall mean and declined similarly on both sides of the centre peak were deemed undesirable. The residuals in Fig. 5b were found to be dispersed and devoid of any discernible pattern, demonstrating the adequacy of the model. Figure 5c depicted a random distribution of data points around the central line, as shown by the studentized residuals and run numbers plot. The randomization procedure assures that the results of the study are not biased by any patterns that may appear. By transforming the data for normalization, the Box-Cox plot of the model transformation approach may assist verify data that aren't regularly distributed. The green line in Fig. 5d denoted the best λ-value (2.13), while the blue line signified the transformation ( *λ* = 1), while the red lines reflect the lowest and greatest values of the confidence intervals (1.3 to 3.05). Because the optimal value was within the bounds of the confidence intervals, no proposal for data transformation was necessary. According to Fig. 5e, the distribution of experimental points seems to be evenly distributed, indicating a normal distribution. A regression model that accurately captures the data acquired, reflecting nearly 97% of the variability of response is thus developed. The adequacy of the model was further evaluated using residuals. Finally, these model diagnostic plots showed that all of the assumptions for the fit-model that predicted the response were met. This means that the model was well-fitting overall.

### Optimization using desirability function (DF)

The goal of the experimental design is to determine the most ideal expected circumstances that will result in the greatest amount of responses possible. To do this, the desirability function (DF) option was used. Spots where the desirability function increases are identified via the mathematical optimization procedure [36]. Response optimization was used to figure out the most efficient combinations of the parameters under study that promote the production of ALP by *B. licheniformis* strain ALP3 to maximize the robustness of ALP productivity. As can be seen in Fig. 5f, which depicts the optimal ALP production optimization plot and its associated desirability function, are the highest predicted values. The highest predicted value of ALP (145 U L^−1^) was obtained when *B. licheniformis* strain ALP3 was grown at 50 °C and 200 rpm for 24 h in media composition of g L^−1^: crab shells powder (46.3), NaNO_3_ (0.3), K_2_HPO_4_ (0.06), ZnSO_4_·7H_2_O (0.0042) and CoCl_2_·6H_2_O (0.0047) without pH adjustment. The model equation was put to the test to see whether it could correctly predict the ideal response value under the suggested optimum circumstances. To ensure that the optimization method's theoretical predictions were correct, laboratory testing was required. Because of the near-perfect agreement between experimental and projected values, it can be said that the DF accurately anticipated the best conditions for *B. licheniformis* strain ALP3 to produce ALP with around 98% accuracy.

### Contour and three-dimensional (3D) plots

**Fig. 6.**
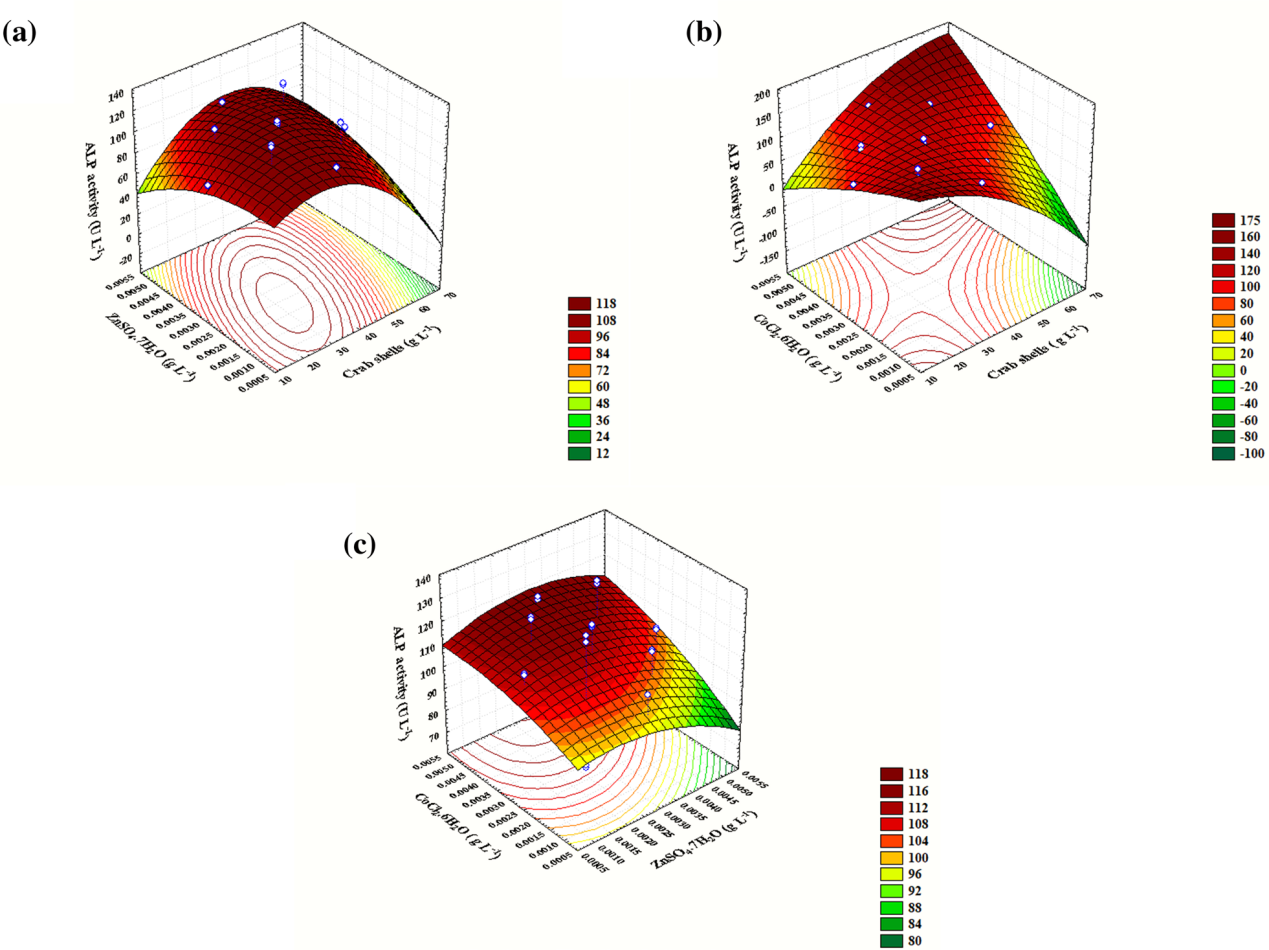
3D response surface representing ALP activity yield (U L^−1^ min^−1^) from *B. licheniformis* strain ALP3 as affected by culture conditions: **a** Interaction between crab shells powder and ZnSO_4_.7H_2_O. **b** Interaction between crab shells powder and CoCl_2_.6H_2_O. **c** Interaction between CoCl_2_.6H_2_O and ZnSO_4_.7H_2_O

Contour plots and surface response plots are two types of diagrammatic representations of response values. These plots are beneficial for visualizing the magnitude of each variable's and interaction's influence. Additionally, it may be used to describe the correlation between independent variables and their associated responses [7, 20]. In this study, three-dimensional plots were constructed using ALP activity (U L^−1^) plotted on the Z-axis against two independent variables of the three following variables (crab shells powder, ZnSO_4_·7H_2_O, and CoCl_2_·6H_2_O), while the rest of the variables stayed its zero values as shown in Fig. 6. Using a 3D surface map, it was possible to demonstrate that crab shell powder (*X*_*1*_) and ZnSO_4_·7H_2_O (*X*_*2*_) concentrations affected ALP production at 0.003 g L^−1^ of zero-level CoCl_2_·6H_2_O (*X*_*3*_) concentration (Fig. 6a). To get ALP activity going, crab shell powder (*X*_*1*_) and ZnSO_4_·7H_2_O (*X*_*2*_) concentrations had to be near their axial points (33.69 and 0.0026 g L^−1^, respectively), and then, ALP production started to drop. The highest ALP throughput (123.30 U L^−1^) was supported at a level where both variables were close to their ideal concentrations. Any change in these levels would have a negative effect on productivity. When CoCl_2_·6H_2_O (*X*_*3*_) concentration was increased to its optimal point (0.0047 g L^−1^) and ZnSO_4_·7H_2_O (*X*_*2*_) was kept at its zero levels, the crab shells powder (*X*_*1*_) boosted ALP production (142.43 U L^−1^) when its concentration was above its centre point (52.128 g L^−1^), as shown in Fig. 6b. Increasing both concentrations above their recorded points, on the other hand, resulted in a decrease in ALP productivity. According to the findings of Fig. 6c, it was discovered that when the crab shell powder (*X*_*1*_) concentration was kept constant, the ALP production efficiencies increased linearly with increasing CoCl_2_·6H_2_O (*X*_*3*_) concentration to 0.00387 g L^−1^, reaching their maximum efficiency (125 U L^−1^). However, the lowest concentration of ZnSO_4_·7H_2_O (*X*_*2*_) was discovered near its centre point (0.0028 g L^−1^), which assisted in accelerating the production process. ALP production declines stepwise way as the concentration of ZnSO_4_·7H_2_O (*X*_*2*_) continues to grow. Three-dimensional (3D) and contour plots show that there is no significant correlation between any two factors, and the acquired data show that they had minimal impact on improving the overall output of ALP.

### Scale-up fermentation strategies for *B. licheniformis* strain ALP3 ALP

An important step in ensuring the profitability of a bioproduct is the scaling up of the process. Before implementing a bioprocess on a large scale, it is common to practice conducting batch cultivations to fine-tune the parameters. To successfully produce ALP in industrial fermenters, effective scaling up is a need. Cell kinetics and bioreactor fluid dynamics, as well as their interplay, are a never-ending work and challenge for biotechnological research, to speed up the transition from laboratory study to industrial application [12, 37]. The goal of this study was to determine whether there is a relationship between the rate of ALP production and the specific growth of a culture. *B. licheniformis* strain ALP3's cell growth kinetics and ALP production properties are shown in Table 5 using various cultivation approaches. The most often used cell growth kinetic model was utilized to calculate the maximal specific cell growth rate [38].

**Table 5:** Kinetic parameters of cell growth and ALP production by *B. licheniformis* strain ALP3 as affected by different cultivation strategies

Parameters	Shake flask cultivation	Uncontrollable pH Batch Cultivation	Controllable pH Batch Cultivation
Growth parameters			
${ X}_{max}$ (g. L^−1^)	1.880	2.625	2.52
µ (h^−1^)	0.131	0.343	0.381
${ \mu }_{max}$ (h^−1^)	0.403	0.695	0.489
$\frac{ dX}{dt}$ (g. L^−1^. h^−1^)	0.112	0.238	0.250
Production parameters			
${ P}_{max}$(IU L^−1^)	178.56	212.29	186.41
${P}_{max. specific}$ (IU g^−1^)	28.034	31.165	28.533
${ P}_{max. time}$ (h)	30	22	28
${ Q}_{p}$ (IU L^−1^ h^−1^)	5.961	10.824	7.330
-${Q}_{s}$ (g L^−1^. h^−1^)	0.013	0.018	0.016
Yield coefficient parameters			
${ Y}_\frac{p}{s}$ (IU g^−1^)	182.7	795.4	688.9
${ Y}_\frac{p}{x}$ (IU g^−1^)	49.8	39.3	40.7
${ Y}_\frac{x}{s}$ (g g^−1^)	1.5	15.8	15.3
Overall cultivation time (h)	32	32	32

*X*_*max*_, maximal cell dry weight; $\frac{dX}{dt}$, cell growth rate; *µ*, specific growth rate; *P*_*max*_, maximal ALP production; *P*_*max specific*_, specific productivity; ${Q}_{p}$, ALP production rate; ${Q}_{s}$, substrate consumption rate; ${Y}_\frac{p}{x}$, U g^−1^ of ALP produced per g biomass; ${Y}_\frac{p}{s}$, U g^−1^ of ALP produced per g substrate consumed; ${Y}_\frac{x}{s}$, g g^−1^, of biomass produced per g substrate consumed

$$\mu =\frac{{\mu }_{max}}{{K}_{s}}+S$$

where μ rate of specific cell growth (h^−1^), ${\mu }_{max}$ maximum specific cell growth rate (h^−1^), the concentration of S substrate (g L^−1^), and ${K}_{s}$ saturation constant. The yield coefficient was determined using the formula;

$${{Y}_{{}^{x}/{}_{s}}}=\frac{\Delta X}{\Delta S}$$

A correlation between specific productivity and specific growth rate per process time is known as the production kinetics, and it can be computed in the same way for batch and fed-batch fermentation systems, as shown in the following formula [39]:

$$\mu t=\frac{\mathrm{ln}\left(X*V\right)-\mathrm{ln}\left({X}_{0}*{V}_{0}\right)}{\left(t-{t}_{0}\right)}$$

where, $X$ is a cell concentration, ${X}_{0}$ is an initial cell concentration, $V$ is the final broth volume and ${V}_{0}$ are the initial broth volume at measuring time $t$ and initial time ${t}_{0}$ respectively. Understanding the growth and production dynamics of *B. licheniformis* strain ALP3 is the goal of this experiment.

### Cell growth kinetics and ALP production in shake-flask under batch conditions

**Fig. 7:**
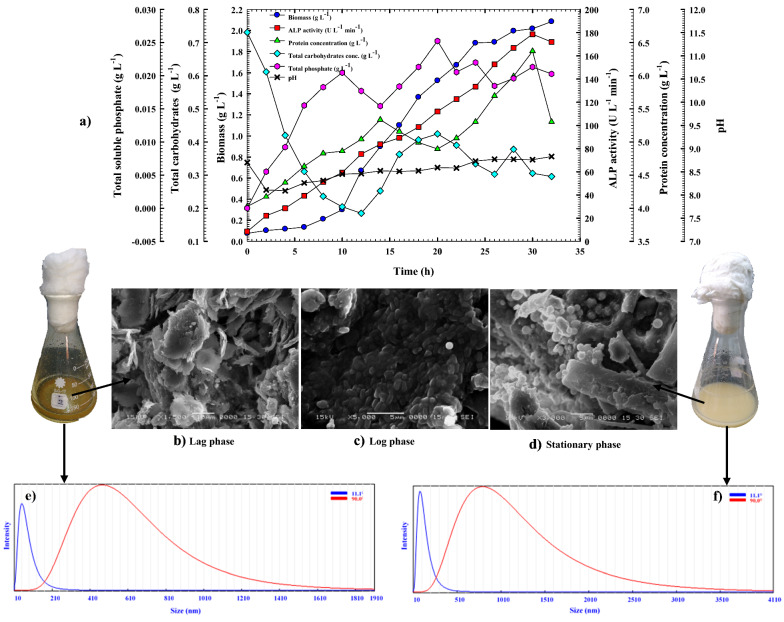
Monitoring of *B. licheniformis* strain ALP3 growth and ALP productivity in (**a**) A shake-flask scale cultivation condition. **b** Scanning Electron Microscope (SEM) micrograph of *B. licheniformis* strain ALP3 during lag phase, log phase, and stationary showing the morphology of the cell during the growth and crab shells particles. **c** Bacterial culture growth of *B. licheniformis* strain ALP3 at zero incubation time. **d** Bacterial culture growth of *B. licheniformis* strain ALP3 at the end of the fermentation process. **e** Particle size distributions analysis of crab shells particles at zero incubation time. **f** Particle size distributions analysis of crab shells particles at the end of the fermentation process

It is possible to batch culture microorganisms in parallel at minimal cost by using shake-flask culture, which is aerobic and attempts to give enough stirring and oxygen to the culture broth. Shake flasks have been extensively utilized in the research and optimization of biotechnology processes because they allow for the conduct of experiments with the least amount of resources and material [40]. Since secondary metabolites are routinely screened and microbe growth conditions are optimized using shake-flask culture, it is a common technique in the early stages of bioprocess development. Shake flasks, unlike agitated bioreactors (such as jar fermenters), do not have specific instruments for monitoring the different culture conditions [40, 41]. A bioreactor-scale process is thus necessary for the production of large quantities of the final product from shaking flasks. *B. licheniformis* strain ALP3 on an optimized medium in a shake flask under standard cultivation conditions was monitored for the production of extracellular ALP as part of this study. When grown in shake flasks, this medium composition also influences the cultivation period required to attain the maximum biomass and ALP production by this strain. It is obvious from Fig. 7a that the *B. licheniformis* strain ALP3 grew appropriately, and ALP was produced concurrently with cell growth. After a lag period (8 h), cells proliferated exponentially, cells grew at a rate of 0.112 (g L^−1^ h^−1^) and a specific growth rate (*µ*) of 0.131 h^−1^. The lag phase, which is essential for the cells to acclimatize to the fermentation medium and conditions, begins after this time. The logarithmic phase displays an increase in biomass and a directly proportionate increase in microbe concentration [38]: $\mathrm{ln}x=\mathrm{ln}{x}_{0}+\mu t$. It was noticed that at 24 h of incubation time, biomass production (1.880 g L^−1^) peaked with a noteworthy yield coefficient *Y*_*x/s*_ (1.5 g g^−1^), driving cell development towards the stationary phase. The cells grow more slowly at the beginning of the cultivation period, which provides them more time to produce ALP as the period progresses. It was discovered that the ALP encoding genes were expressed gradually from the beginning of the cultivation to its crest (179.56 U L^−1^) at which point the production rate (*Q*_*p*_) was 5.96 U L^−1^ h^−1^ at the late time of the stationary phase (30 h) of *B. licheniformis* strain ALP3 growth pattern. After the production curve hit this point, it started to decline. An additional step in deciphering the performance of ALP-producing cells was to construct yield coefficients for that enzyme (*Y*_*p/s*_, and *Y*_*p/x*_) as well as its specific productivity (*P*_*max specific*_). According to this study, *Y*_*p/s*_, *Y*_*p/x,*_ and *P*_*max specific*_ were all 182.7, 49.8, and 28.034 U g^−1^, respectively. This finding contradicts the findings of other investigators who detected an increase in phosphatase production during exponential growth of a *Citrobacter* sp. [42], *B. paralicheniformis* strain APSO [7], and *Lysinibacillus* sp. APSO [20].

However, the protein content climbed progressively from the commencement of bacterial growth to 5.33 g L^−1^ at 14 h, and then decreased slightly to 4.99 g L^−1^ at late of the log phase (20 h), before increasing again to reach its pinnacle (6.36 g L^−1^) at 30 h, which is similar to the peak time of ALP production. Beyond this time, a sharp decline in protein content was observed. Because the carb shells powder was used as the sole source of carbon in the medium ingredients, which comprise between 20 and 30% chitin [5], the growth of *B. licheniformis* strain ALP3 on the other hand, was associated with carbohydrate consumption and/or production, resulting in a fluctuating pattern throughout the fermentation period. Cell growth caused the total carbohydrate content to plummet gradually from the initial concentration of 0.730 to 0.204 g L^−1^ after 10 h of the cultivation process, but it climbed back up gradually to 0.424 g L^−1^ at 20 h of cultivation because the chitinase enzyme broke down the chitin portion, which led to an increase in the total carbohydrate concentration. This was followed by another decline trend before an increase in the total carbohydrate concentration again. ALP and organic acid production by *B. licheniformis* strain ALP3 resulted in the release of inorganic P from crab shells particles into the culture medium, which was then taken up as a nutrient for bacterial growth, according to the findings of the phosphate content pattern tracking. As can be seen in Fig. 7a, phosphate content fluctuates throughout the fermentation process. According to this pattern, the upward peak of phosphate content was a result of the release of inorganic phosphate from crab shells particles (upward peak), and the downward peak was due to the consumption of inorganic phosphate by bacterial cells. It is clear from Fig. 7a and b that the phosphate content increased steadily throughout the course of the incubation period, reaching as high as 0.020 g L^−1^ after 10 h of incubation time before bacterial consumption of released inorganic phosphate dropped it to 0.015 g L^−1^ at 14 h of the fermentation process. Afterwards, with proceeding in the log phase, the inorganic phosphate content gradually increased again until it reached a peak of 0.025 g L^−1^ after 20 h of incubation as a result of the bio-solubilization process, and then plateaued with minor fluctuations until the end of the fermentation process. Inorganic phosphate upward peaks at 24 and 30 h coincided with the peak time of *B. licheniformis* strain ALP3 growth and ALP production, according to the study. Changes in pH were shown to be correlated with the solubilization and production of ALP. *B. licheniformis* strain ALP3 culture medium had a pH of 8.69 at the beginning of incubation time, which gradually decreased to 8.09 over the first four h, then steadily rose again during the fermentation process until it reached 8.83 at the end of the cultivation period as illustrated in Fig. 7a. Protein content and ALP production both increased in tandem with bacterial growth when the pH shifted. These findings were attributable to the pH-dependent control of gene expression for enzyme production as explained by Qureshi et al. [25].

### Heavy metals survey

Atomic absorption spectrometry (AAS) was used to identify heavy metals in the crab shells particles that were leftover following the shake-flask cultivation process. During the fermentation process, *B. licheniformis* strain ALP3 can solubilize transition and alkali metals from the crab shell particles owing to the production of phosphatase enzyme and organic acid. There was a significant increase in Co^+^, K^+^, Fe^+^, Cd^2+^, Cr^+^, Zn^+^, Mn^2+^, Ca^2+^, and Na^+^ concentrations in shake-flask-cultivated crab shell samples compared to their initial concentrations before fermentation at zero incubation time by a factor of 170.34, 2.45, 2.39, 1.24, 2.8, 1.69, 1.61, 3.32, and 2.37; respectively, this was due to the leaching and accumulation of these elements during the fermentation process as cited in Table 6. However, the growth of *B. licheniformis* strain ALP3 was found to be accompanied by a decrease in Ni^+^, Mg^+^, Li^+^, and Cu^2+^ concentrations, when compared to that detected in the crab shell leftover sample at zero time incubation by factors of 283, 1.23, 1.19, and 1.59, respectively. As a result of this discovery, it has been demonstrated that such metal ions are essential in both growth and ALP production. Chaudhuri et al. [43], who demonstrated the importance of metal ion chelation for enzyme production or stability, concur with this finding. Figure 7b shows SEM micrographs of the crab shells leftover sample taken at zero incubation time showing a smooth surface with particle sizes of 497.6 and 56.1 nm at angles of 90 and 11°, respectively, as illustrated in Fig. 7e. The number of bacterial cells was increased during the log phase by the division of multiplied cells into a tiny size endospore-forming bacilli cell, which strongly clung to the crab shell's surface as seen by SEM micrographs (Fig. 7c). At the end of the growth phase, the bacterial biomass continued to proliferate although ALP production, protein concentration, and total carbohydrate concentration decreased. It was discovered that bacterial cells were transformed into spore cells, as illustrated in the SEM micrograph (Fig. 7d), which clarifies the shape of spore cells, and the cell spores were still stuck to the crab shell surface. As illustrated in Fig. 7f, the particle size of crab shells left behind after the fermentation process was calculated to be 885.6 nm at a 90° angle and 99.6 nm at an 11° angle. An increase in the size of crab shell particles is evident compared to that recorded at zero incubation time by 1.77 times, and it is caused by bacterial cells adhering to the shell surface during fermentation processes.

**Table 6:** Flame Atomic Absorption Spectrometry for heavy metals analysis of crab shells leftover samples at zero incubation time and after 32 h under shake flask cultivation condition

Metals conc. (mg/Kg)	Crab shells leftover sample at zero incubation time	Crab shells leftover sample after 32 h incubation time
Co	0.0317	**5.40**
K	5430	**13,335**
Fe	211.7	**507.1**
Ni	5.93	< 0.02
Cd	12.25	**15.29**
Cr	5.66	**15.85**
Zn	49.81	**84.40**
Mg	14,160	11,445
Mn	44.26	**71.27**
Li	7.12	5.94
Ca	212,400	**707,250**
Na	20,730	**49,215**
Cu	22.84	14.345

Significant values are indicated in bold

### Cell growth kinetics and ALP production in the bioreactor under uncontrolled pH batch conditions

**Fig. 8:**
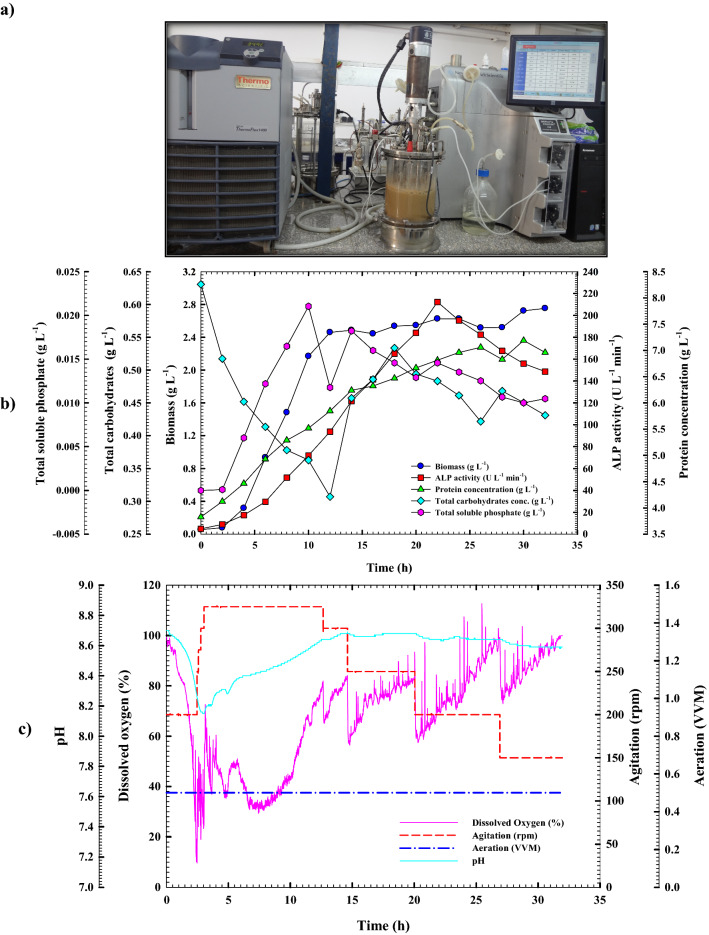
Monitoring of *B. licheniformis* strain ALP3 growth and ALP productivity in **a**. A 7 L stirred-tank bioreactor cultivation system. **b** Uncontrolled pH growth cultural conditions. **(c)** Online data (DO, agitation, aeration, and pH) as a function of time during batch fermentation in the bioreactor under uncontrolled pH conditions

The move from small-scale optimization to large-scale industrial production of a fermentation process is a vital step in the successful transfer of technology and commercialization of a product of interest. Both simple and sophisticated bioreactors may be utilized to achieve effective fermentation. The bioreactor, considered the beating heart of all bioprocess activities, is where nutrients are converted by microbial cells into the desired metabolites [37, 40]. So far, stirred-tank reactors (STRs) have been the most common fermenters used in the lab to study submerged fermentation (SmF) on a small scale. As a result, *B. licheniformis* strain ALP3 was grown in a 7 L stirred-tank bench-top bioreactor (Bioflo 310; New Brunswick Scientific, Edison, NJ, USA) at an uncontrolled pH condition for further optimization and augmentation of ALP production as illustrated in Fig. 8a. All of the patterns produced in this investigation were remarkably analogous to those discovered in the shake-flask counterpart, as shown in Fig. 8b. When the dissolved oxygen concentration was maintained above 20% by increasing the agitation rate from 200 to 350 rpm throughout the incubation period, it was discovered that the ALP production rate was significantly increased and boosted, with the ALP's time to production being reduced by approximately 8 h when compared to its shake flask counterpart. In terms of scaling up, modelling, and controlling, growth kinetic models are one of the most significant models. From simple exponential growth to complicated mathematical expressions, the kinetic model has developed to predict heterogeneity in single cells, describe numerous processes, explain internal control systems, and even predict genetic diversity within bacterial populations. Thus, the design of operating conditions and operational design for optimum product production was made possible by this simulation model [38, 39]. Through the bioreactor cultivation system, the ALP production capacity, rate of production, and specific productivity are all much more than those obtained using shake flask cultivation. The trend of cell proliferation was paralleled by the volumetric ALP production and protein content patterns. At the mid-stationary phase (22 h) of *B. licheniformis* strain ALP3 growth, the highest peak of ALP production with the greatest ALP-specific production (31.165 U g^−1^) and the greatest production rate *Q*_*p*_ (10.824 U L^−1^ h^−1^) was recorded as 212.29 U L^−1^, and the production curve afterwards started to flatten out. The observed findings surpassed those reported for the shake flask model in terms of production capacity, specific productivity, and production rate by 1.188, 1.111, and 1.815 times, respectively. There was a substantial increase in the yield coefficients *Y*_*p/s*_ of ALP, which rose to 795.4 U g^−1^ by a factor of 4.35 above those obtained in the shake flask mode, as a result of these discoveries. In addition, the increase in ALP production was associated with a trend toward increasing protein content; the protein content curve started to go down with some fluctuations after it hit its peak point at 26 h, which was 7.057 g L^−1^. When *B. licheniformis* strain ALP3 is grown in a stirred-tank bioreactor, it exhibits growth patterns that are strikingly similar to those seen in a shake flask. The bacterial cells grew exponentially over time with no discernible phase lag; the highest cell biomass production (*X*_*max*_; 2.45 g L^−1^) with a growth rate of 0.238 g L^−1^ h^−1^ and a specific growth rate (*µ*) of 0.343 h^−1^ was attained after 12 h of incubation time, which was approximately 12 h earlier than in the shake-flask. After crossing its peak point, the stationary phase began at 14 h of incubation time. An increase of 30.79% in biomass production has been achieved with the significant yield coefficient *Y*_*x/s*_ of 15.8 g g^−1^, which is 10.5 times higher than those reported through the shake flask cultivation mode according to the obtained results. Attributable to excellent aeration and agitation, no substantial lag in growth has been detected, which is most likely due to this. The amount of total carbohydrate in the fermentation medium, which was measured throughout the cultivation process, was shown to be inversely related to ALP3 cell proliferation and ALP production resulting in a fluctuations pattern. As cell growth and ALP production increased, a consumption rate (*Q*_*s*_) of 0.018 g L^–1^ h^–1^ was detected, which was a factor of 1.38 greater than that seen in shake-flask culture. Meanwhile, after 12 h of incubation time, the total carbohydrate concentration decreased noticeably, approaching the biomass peak period, showing that the available total carbohydrate plays a significant role in bacterial growth. As a consequence of bacterial chitinase enzyme activity on crab shell chitin and subsequent consumption by bacterial cells, the total carbohydrate content steadily rose again to 0.53 g L^−1^ at 18 h of incubation time, before falling to 0.42 g L^−1^ at 26 h. As the fermentation process progressed, the total carbohydrate trend fluctuated between high and low levels. Furthermore, phosphate content pattern monitoring revealed, as shown in Fig. 8b, that the phosphate content fluctuated in response to the action of ALP on the calcium phosphate part of crab shells particles and subsequent phosphate consumption by bacterial cells which looks like that obtained through shake flask cultivation mode. The inorganic phosphate concentration reached its peak of 0.021 g L^−1^ as it approached the biomass peak time (10 h) and then dropped to 0.011 g L^−1^ for the next two further hours when it hit the biomass peak time. The inorganic phosphate content trend varied between high and low values as the fermentation process moved toward the stationary phase. The pH of the culture medium followed a pattern that was quite similar to that of the shake flask counterpart. As illustrated in Fig. 8c, during the first three hours of incubation time, the pH of the *B. licheniformis* strain ALP3 culture medium was gradually decreased from 8.7 to 8.15, then steadily rose to 8.61 at 12 h of incubation time which is equivalent to the biomass peak time. After then, the pH of the culture medium stayed relatively steady, ranging from 6.61 to 6.68, with only minor fluctuations throughout the fermentation process.

Throughout the fermentation process, it was noticed that during the early phases of fermentation, the *B. licheniformis* strain ALP3 had a significant decline in DO concentration neared zero% as its biomass rose rapidly, this, in turn, caused a litter titter of ALP produced (data not shown). Many *Bacillus* spp. have been shown to produce enzymes that are influenced by the quantity of dissolved oxygen. Consequently, forcing the fermentation medium to aerate might be fruitful, since combining aeration with stirring increases efficiency [41]. Therefore, the availability of dissolved oxygen (DO) in the growth media was maintained at above 20% by increasing the agitation rate throughout the cultivation period in this study. As illustrated in Fig. 8c, after 2 h of incubation, the dissolved oxygen concentration had declined to 13.6%; as a result, the agitation rate was gradually raised from 200 to 325 rpm during the fermentation process, and the dissolved oxygen concentration rose as a result. It was noticed that the dissolved oxygen content dropped steadily throughout the first 12 h of the fermentation process, therefore the agitation rate was automatically raised from 200 to 325 rpm during this time to provide more oxygen to the culture medium, which corresponded to the biomass rising trend. After surpassing this point, the dissolved oxygen concentration was gradually raised again until it reached 74%. As a result, the agitation rate was automatically reduced from 325 to 150 rpm to maintain an appropriate percentage of dissolved oxygen. The current findings demonstrate that adjusting the agitation speed and aeration rate in the fermenter may significantly increase gas–liquid transfer and mixing in the fermenter, hence increasing the amount of ALP produced. According to the authors' knowledge, this is the first report on boosting ALP yield by process optimization of bioreactor operational factors.

### Cell growth kinetics and ALP production in the bioreactor under controlled pH batch conditions

**Fig. 9:**
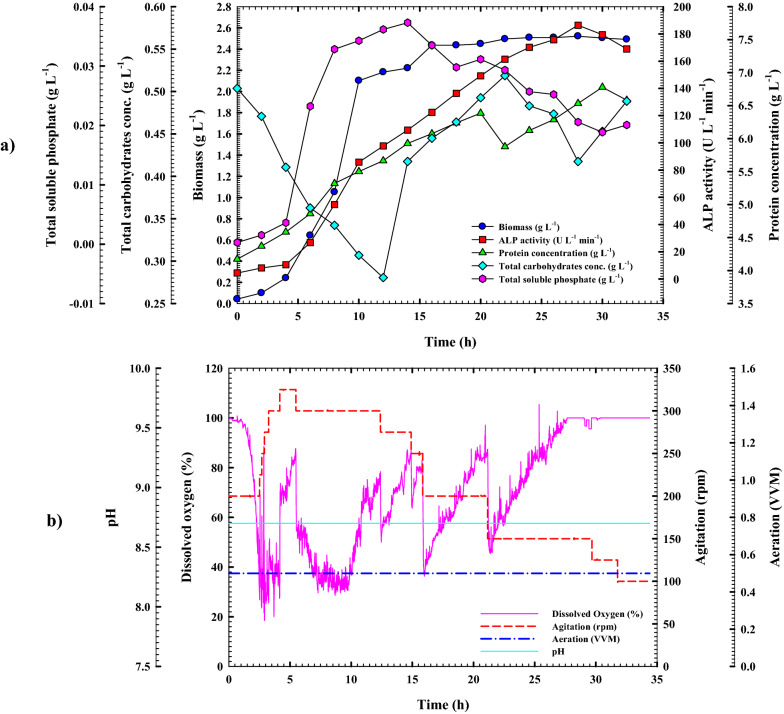
**a** Monitoring of *B. licheniformis* strain ALP3 growth and ALP productivity in a 7 L stirred-tank bioreactor under controlled pH conditions. **b** Online data (DO, agitation, aeration, and pH) as a function of time during batch fermentation

Aside from the above, *B. licheniformis* strain ALP3 was grown in a 7-L stirred-tank bench-top bioreactor at a constant pH of 8.77 settings to better understand the influence of pH on the production process. According to the findings of this experiment, the growth profiles of cells, volumetric ALP production profiles, and substrate consumption profiles have been shown in Fig. 9. As with prior culture modes, the bacterial cells grew exponentially over time, with no discernible phase lag. With a growth rate of 0.250 g L^−1^ h^−1^ and a specific growth rate (*μ*) of 0.381 h^−1^, the cell growth increased gradually over 16 h of the incubation period, reaching its maximum (*X*_*max*_ = 2.52 g h L^−1^). Although ALP3 growth was significantly earlier than those achieved in shake-flask cultivation systems by 14 h, it was delayed by 4 h compared to those recorded under an uncontrolled pH condition. Thus, a 15.315.3 g g^−1^ yield coefficient *Y*_*x/s*_ was reported, which was substantially higher than the yield coefficients achieved through shake flask cultivation mode by a factor of 10.2, but it was approximately 1.03 times less than the yield coefficients obtained through uncontrolled pH cultivation mode. This study emphasizes the crucial function of raising the agitation rate throughout the fermentation process to modify the dissolved oxygen concentration to a desirable level. Although it shifted to become earlier than those obtained did shake flask cultivation system. ALP productivity was 6 h delayed when compared to results obtained with an uncontrolled pH system, where ALP volumetric productivity increased steadily over the course of the cultivation time with a production rate (*Q*_*p*_) of 7.33 and peaked at 186.41 U L^–1^ after 28 h.

Along with this, it was shown that the volumetric productivity of ALP dropped by 13.88%, resulting in a reduction in the yield coefficient *Y*_*p/x*_ (688.9 U g^–1^) by a factor of 1.15 as compared to an uncontrolled pH cultivation system. But volumetric ALP production increased by 4.39%, leading to a 1.15 times increase in the yield coefficient *Y*_*p/x*_ when compared to the shake flask cultivation mode. In addition, the protein content trend in the above-mentioned culture conditions was also in lockstep with the growth and ALP production patterns, rising steadily throughout the fermentation until it reached a peak concentration of 6.38 g L^–1^ at 20 h. This was followed by a two-hour decline trend before climbing back up again and reaching its peak of 6.77 g L^–1^ at 30 h of incubation time as illustrated in Fig. 9a. A gradual decrease in ALP production and protein content trends was seen after this time. Furthermore, it was shown that substrate consumption was associated with growth and ALP production. Since substrate consumption increased in tandem with cell growth and enzyme production, with a consumption rate of 0.016 g L^−1^ h^−1^, the total carbohydrate concentrations fell steadily during the fermentation process, reaching 0.28 g L^−1^ after 12 h of incubation before rebounding again to 0.518 g L^−1^ at 22 h of incubation time. Then, at 28 h of incubation time before the last rise towards the end of the fermentation process, there was a drop of 0.41 g L^−1^. Furthermore, as illustrated in Fig. 9a, the inorganic phosphate concentration peaked at 0.037 g L^−1^ as the fermentation process approached the biomass peak time (14 h) and then gradually decreased as the fermentation process progressed toward the stationary phase, eventually dropping to 0.018 g L^−1^ at the end of the fermentation process. When comparing uncontrolled pH batch and shake-flask cultivation systems, it was discovered that the substrate consumption and phosphate content patterns were quite similar. Additionally, the concentration of dissolved oxygen (DO) in the growth medium was kept above 20% under control conditions, as was the case with uncontrolled pH cultivation. With just two h of incubation time left, the dissolved oxygen concentration had plummeted to 20%; as a consequence, the agitation rate was raised by an automated process from 200 to 325 rpm, and the dissolved oxygen concentration subsequently climbed as shown in Fig. 9b. Dissolved oxygen percentage rose to 86.5% after 5 h of fermentation process as a consequence of controlled pH conditions, which were shown to occur 7 h earlier than under uncontrolled pH settings. It was thus necessary to automatically reduce stirring speeds from 325 to 150 rpm to keep the concentration of dissolved oxygen within acceptable limits. Finally, according to the findings of this research, *B. licheniformis* strain ALP3 was no longer able to produce ALP as efficiently when grown in pH-controlled conditions due to the adverse effects of a constant pH on growth and ALP production, this result corroborated the results of Abdelgalil et al. [7, 20]. ALP productivity is best promoted and boosted under an uncontrolled pH cultivation condition mode, as a consequence. By using simple and low-cost ingredients for remediation of crab shell waste in line with ALP production, the innovative formulation medium for ALP production has been produced via the current investigation. The production of ALP from *B. licheniformis* strain ALP3 was optimized in stages, beginning with PBD (90.06 U L^−1^), then to SCCD (111.6 U L^−1^), and lastly to uncontrolled pH batch culture technique designs (212.29 U L^−1^), until eventually reaching the optimal level. When compared to the initial media, productivity increased by 1.21, 1.51, and 2.87 times, respectively, throughout the process. However, the highest improvement of ALP production from *B. paralicheniformis* strain APSO was systematically achieved through PBD, Rotatable CCD, and an uncontrolled pH batch cultivation strategy by factors of 20.96, 70.12, and 94, respectively, when compared with the basal medium using molasses as agro-industrial waste to induce the production process as documented by Abdelgalil et al. [7]. Moreover, in another study, Abdelgalil et al. [20] recorded that the *Lysinibacillus* sp. strain APSO ALP throughput was enhanced by 9.3-, 16.5-, and 20.75-fold, compared to the initial medium by utilizing the sequential optimization strategy, which started with PBD, followed by CCD-uniform precision, and ended with uncontrolled pH batch cultivation strategy designs; respectively. In Abdelgalil et al. [7, 20], findings, the ALP yield was significantly greater than that in the current study. This is because of the complexity of crab shells structure, which required a large number of enzymes to work cooperatively to solubilize the crab shell. This caused ALP to be produced in small amounts compared to Abdelgalil et al. [7, 20].

### Morphological structure of crab shells particles

**Fig. 10:**
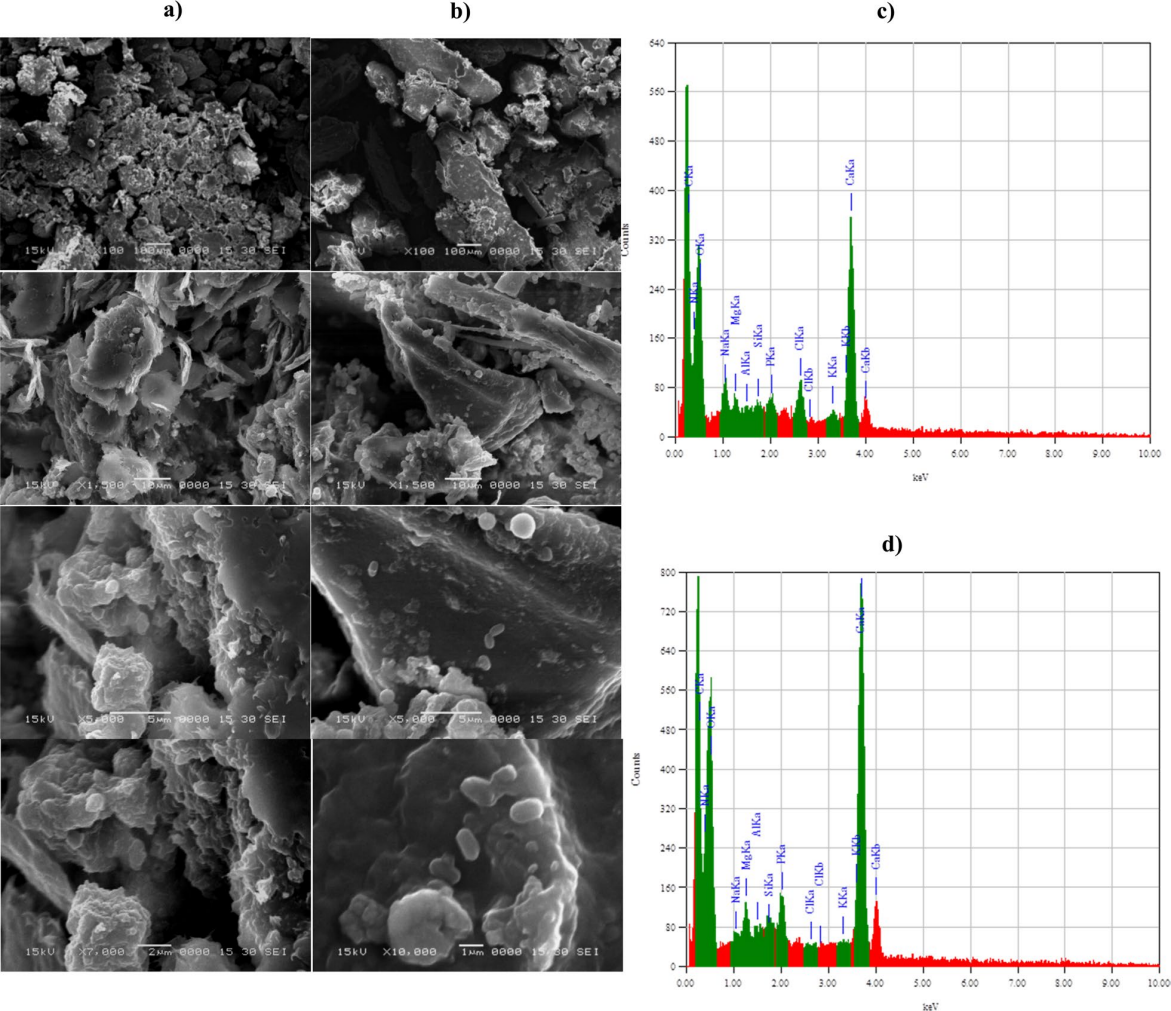
Scanning electron microscopy (SEM) micrograph at a magnification of ×100, ×1500, ×5000, and ×10,000  for the crab shells leftover samples of **a** zero incubation time; **b** After 32 h of incubation time. **c** EDS analysis at zero incubation time. **d** EDS analysis at the end of the fermentation process

After the fermentation was completed, the residual crab shell leftover samples were collected through filter paper and dried overnight at 60 °C in the oven. The powdered crab shells were then subjected to SEM and EDS analysis to better understand their dispersion and surface texture. A technique known as electron dispersive spectroscopy (EDS) uses the amplitude of the x-ray released when an electron is struck by an electron beam to determine whether or not certain elements are present. Throughout the fermentation process, *B. licheniformis* strain ALP3 utilized crab shells powder as a nutrient source for growth and ALP production, resulting in morphological and metal element content changes in the collected sample, as shown in Fig. 10. The surface appearance and microstructure of crab shell samples are visualized using SEM. According to the SEM micrograph (Fig. 10a), the forms and shapes of the crab shell particles are very agglomerated and irregular particles during zero-time incubation and the surface is cracked, rough, and porous. Additionally, the distribution of particle sizes is not uniform, as can be shown in the SEM micrograph (100×). Crab shells particles may be shown to have a densely stacked, layered structure with a few irregularly distributed grooves. Moreover, the crab shell particle micrograph showed layers of crumbling flakes of chitin with a smooth surface appearance and granular structure as illustrated in magnification of 1500 and 5000×. As previously stated, the observations of Knidri et al. [44] were in accord with this finding. CaCO_3_, chitin, and Ca_3_(PO_4_)_2_ are the primary components of the crab shell [45]. EDS of the crab shells particles, as seen in Fig. 10c, reveals that the particles are made up of calcium (Ca), phosphorus (P), silica (Si), aluminium (Al), magnesia (Mg), oxygen (O), Chlorine (Cl), Sodium (Na), potassium (K), and carbon (C). The absorption peaks associated with carbon (AT% of 38.83 and mass % of 24.04), oxygen (AT% of 36.82 and mass % of 30.36), and calcium (AT% of 16.36 and mass % of 35.59) are among the most predominant in the sample, and P (AT% of 0.74 and mass % of 1.18) has a lower abundance, as shown by the absorption peak. These constituents demonstrate that the crab shell particles are mostly made of calcium carbonate (CaCO_3_) in the form of calcite, which is consistent with previous research. As a result, the calcium to phosphorus weight ratio of the crab shell hydroxyapatite was 22.1. On the other hand, as can be shown in Fig. 10b, the morphology of powdered crab shells has altered after 32 h of cultivation time. At 100× magnification, a fluffy irregular particle structure can be seen, which was becoming bigger, as the structure becomes more scattered. One of the most interesting findings from this study was that the crab shell particles had bacterium cells firmly attached to surface-degrading chitin flakes with porosity and grooves, as seen at a magnification of 1500, 5000, and 10,000× Fig. 10b. The element map obtained by the EDX analysis, on the other hand, revealed that the considerable concentrations of oxygen (AT% of 46.27 and mass % of 34.05), followed by calcium (AT% of 25.38 and mass % of 46.79), and carbon (AT% of 23.59 and mass % of 13.03), represented the predominant elements in the sample as depicted in Fig. 10d. The solubilization of CaCO_3_ mediated by the action of organic acids and ALP generated by *B. licheniformis* strain ALP3 corresponds to a chemical reaction of ${\mathrm{CaCO}}_{3}\to \mathrm{CaO}+{\mathrm{CO}}_{2}\uparrow$ during the fermentation process was attributable to the significant increase in AT% for Ca and O, which was about 57.63% and 21.5% higher than those at zero incubation time. However, as compared to the values obtained before the cultivation process, the AT% for carbon decreased by 64.6%. This was because the carbon was utilized by bacterial cells for growth and the production of metabolites throughout the cultivation process. As a consequence of the ALP-induced solubilization of crab shell particles, EDS analysis revealed 2.74-fold higher peaks in P absorption (AT% 2.03 and mass% 2.89) than those recorded at zero incubation time. Thus, the hydroxyapatite weight ratio (Ca:P) of the crab shells decreased by 76.8% after the fermentation process, resulting in a Ca:P ratio of 12.5. Moreover, as a result of bio-solubilization and fermentation processes, the powdered crab shells were liberated and leached of some minerals and other substances.

### Thermal analysis

**Fig. 11:**
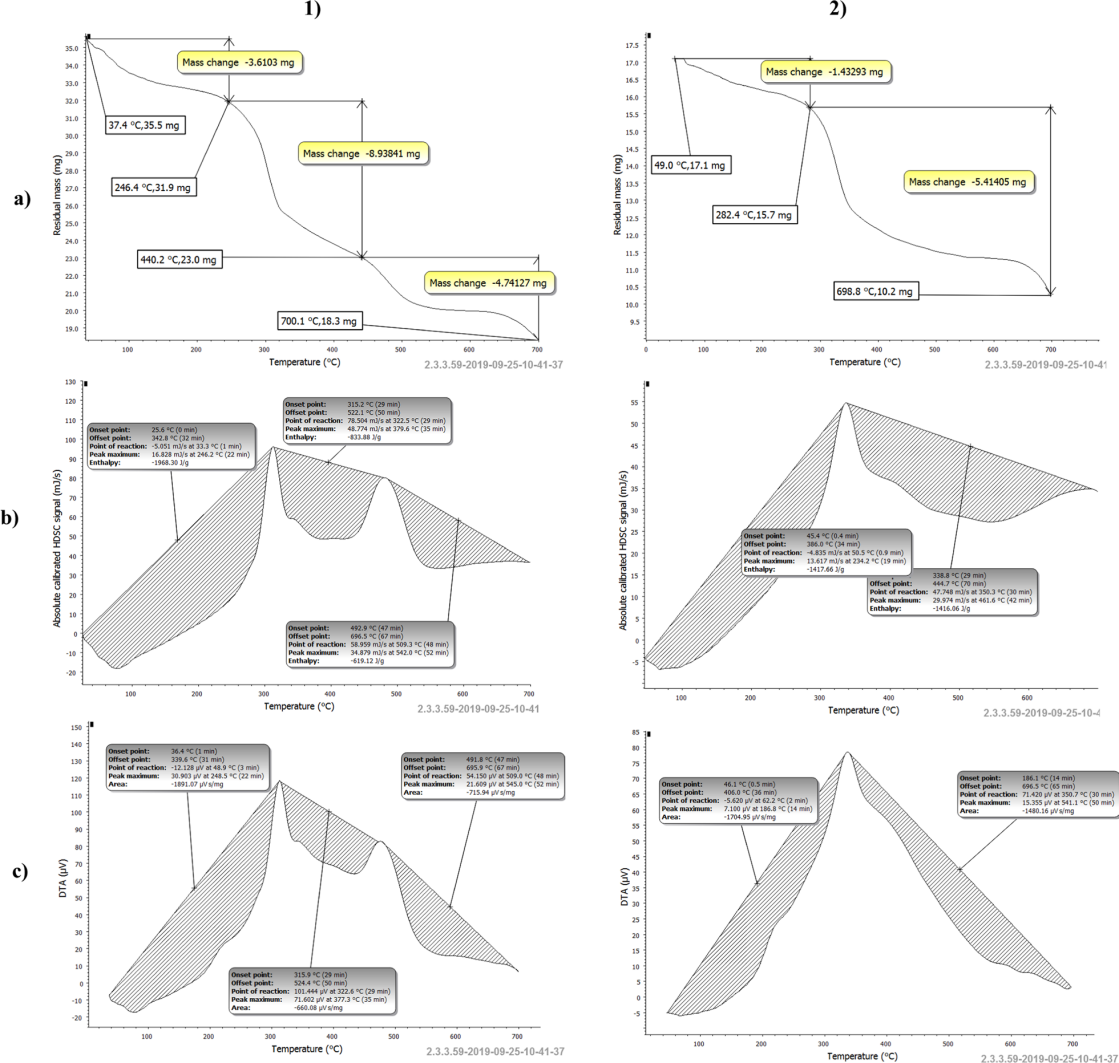
**a** TGA analysis pattern for crab shells leftover samples, **1)** at zero incubation time; **2)** After 32 h of incubation time; **b** DSC analysis pattern; **c)** DTA analysis pattern

For thermal analysis, leftover crab shells collected samples were heated from room temperature to 700 °C at a rate of 10 °C min^–1^ in a liquid nitrogen environment, as shown in Fig. 11. Figure 11a depicts the findings of a thermogravimetric study of crab shells leftover samples after 32 h of incubation time and at zero incubation time to serve as a point of reference. As the temperature rose from room temperature to 700 °C, the curve began to decline, indicating that the weight had been lost. As shown in Fig. 11a1, crab shells left behind at zero incubation time exhibit three different segments of thermal decomposition behaviour. When the temperature reached 246.4 °C, a weight loss of 11.28% and a mass change of − 3.61 mg were observed due to the dehydration of the crab shells samples, which is evident because of the surface moisture and hydrophilicity of chitin constituting crab shells [46]. The second disintegration phase, which occurs at temperatures between 246.4 °C and 440.2 °C, has a high exothermal peak associated with considerable weight loss of 38.69% and a mass change of − 8.93 mg. The depolymerization of chitin, as well as the subsequent loss of CH_2_OH and NH_2_ moieties, may be a major contributor to the observed weight loss [47, 48]. Olajide et al. [49], found similar results in their study with observed weight loss with temperature rise, and this weight loss was also suggested to be probably associated with depolymerization, loss of CH_2_OH, and NH_2_ moieties. All that is left is the decarboxylation of CaCO_3_, which took place between 440.2 and 700.1 °C and resulted in about 25.68% of the shell weight loss, with an accompanying mass change of − 4.99 mg [50]. It was discovered by Sebestyén et al. [51] that the thermal disintegration of the crab sample's main structure reached a temperature of 450 °C. Only sluggish charring processes occurred above this point in the process. According to the results of this study, the studied shells are identical to those of an American lobster known to have a CaCO_3_ biomineral phase as well as chitin-protein fibrils in its shells [52]. After 32 h of incubation time, the TGA patterns of crab shells left behind from the fermentation process were shown in Fig. 11a2 on the other hand. It was discovered that the fermentation process resulted in the presence of two thermal decomposition peaks rather than the three distinct peaks seen at zero incubation time, as had been previously reported. A weight loss of 8.917% was recorded when the endothermic peak appeared at 282.4 °C, which was 2.36 times less than the weight loss observed at zero time. Compared to the zero-time mass change, the mass change was reduced by 2.18 times. The coexistence of an exothermic reaction (for the depolymerization of chitin) and an exothermic reaction (for the decarboxylation of CaCO_3_) caused an area of significant exothermic activity in the temperature range 282.4 to 698.8 °C. Which was resulting in a second and last weight loss of 56.92% with a mass change of 5.41 mg, which was greater than the weight loss reported at zero time incubation by factors of 1.39. This occurred as a result of the fermentation process, which resulted in chitin biodegradation as well as CaCO_3_ bio-solubilization during bacterial growth and ALP production. Moreover, differential scanning calorimetry (DSC) is often recognized as the most effective technology for measuring changes in heat flow throughout the course of a process. There are two possible paths for heat flow: either from the exothermic reaction source to the endothermic reaction source, or a combination of the two. There were three different peaks on the DSC and DTA curves of crab shells leftover samples at zero incubation time similar to those obtained through the TGA pattern as illustrated in Fig. 11b1. The evaporation of water within the structure led the first endothermic peak to appear at 246 °C with a heat flow of 16.82 mJ s^−1^, this caused the enthalpy to be − 1968 J g^−1^. Water-holding capacities of shells increase linearly with endothermic peak temperatures. The Second transition, also known as the main event, is the exothermic peak of the DSC curve at 379.6 °C, which is attributable to N-acetylglucosamine chain cleavage of hydroxyl and acetamide functional groups and represents pyrolytic reactions of polymer chains, mainly the depolymerization of chitin. Calcite thermal dissolution caused an additional exothermic peak at 542 °C, which had an enthalpy of − 619.12 J g^−1^ and an energy flow of 34.87 mJ s^–1^. However, after 32 h of cultivation time, a notable reduction in the number of peaks as well as a shift in heat flow pattern could be seen in the thermal curve (Fig. 11b2). This indicated that the solubilization process had induced a change in the DSC pattern. As a result of this shift, the endothermic peak was found to have appeared at 234.2 °C with a heat flow of 13.61 J s^–1^ and had an enthalpy of − 1417.66 J g^–1^, which was 12 °C earlier than the endothermic peak that had been observed at zero incubation time. Furthermore, the endothermic peak is characterized by a lower heat flow and specific heat capacity than the zero time peak by 3.21 mJ s^–1^ and 552.24 J g^–1^, respectively. Crab shells' physical and chemical properties and structure were altered by the *B. licheniformis* strain ALP3 growth, which resulted in the solubilization of CaCO_3_, calcium phosphate, and chitin depolymerization, which in turn led to the elimination of some thermal peaks through thermal analysis. As a consequence, a single exothermic peak was seen at 461.6 °C, with heat flow and enthalpy values recorded at 29.97 mJ s^–1^ and − 1416.06 J g^–1^, respectively. According to Fig. 11c, the results of the DSC and DTA analyses were reasonably congruent in their interpretation and conclusions.

### Fourier-transform infrared spectroscopy (FT-IR) and XRD analysis

**Fig. 12:**
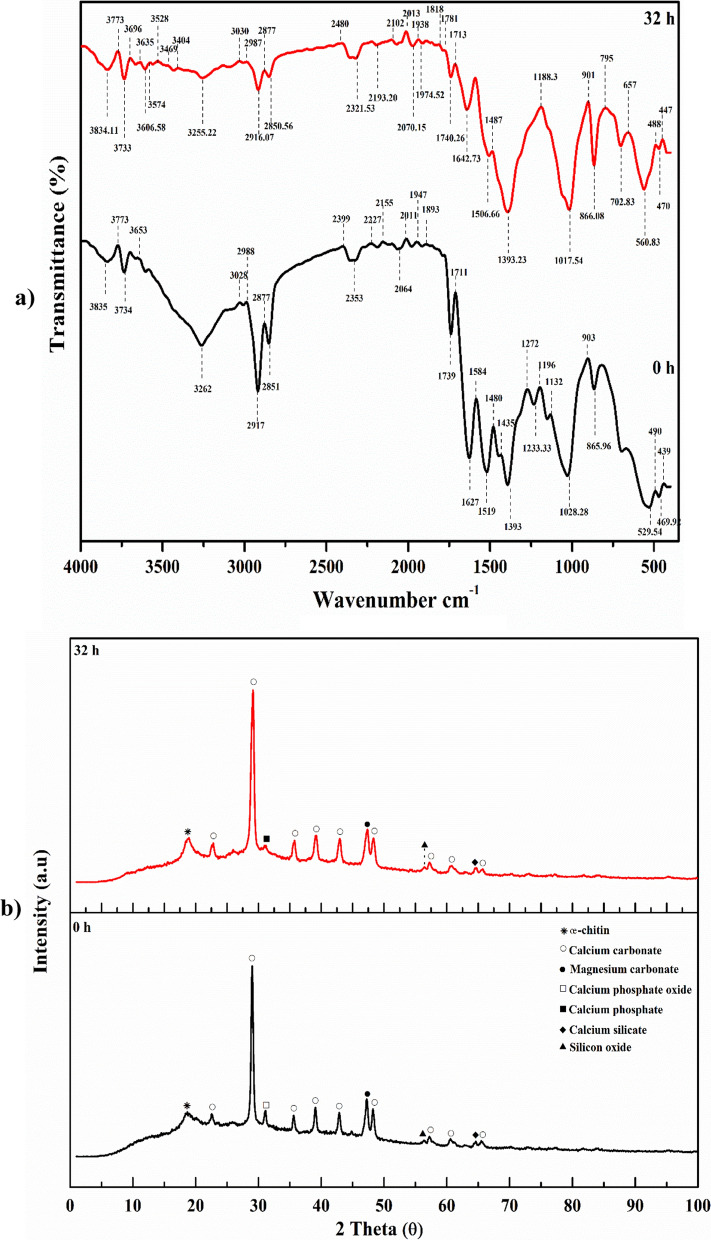
**a** FTIR pattern crab shells residual samples at zero incubation time, and 32 h incubation time. **b** XRD pattern crab shells residual samples at zero incubation time, and 32 h incubation time

Leftover crab shell samples both before and after the fermentation process were submitted to FT-IR analysis, as shown in Fig. 12a, to identify a functional group in the samples and also to assist in the interpretation of the XRD results. There were noticeable and substantial changes in vibrational band strength and position as well as the presence and absence of certain peaks as a consequence of the fermentation and solubilization processes. A major consequence of the solubilization process was a considerable influence on the band intensities, which were altered dramatically. Untreated crab waste exhibited peaks at 1579 cm^−1^, 1622 cm^−1^, 3262 cm^−1^, and 1393 cm^−1^, which are consistent with chitin peaks [53]. According to spectral data, a sharp absorption peaks intensity of crab shells particle, which were taken at zero incubation time, were detected at 3262 and 2353 cm^−1^, which was demonstrated to be typical of α-chitin and corresponded to NH– stretching, which is implicated in intermolecular and intramolecular hydrogen bonds [54]. As a consequence of the chitinase's activity on chitin during the fermentation process, characteristic peaks of chitin were shifted to 3255 cm^−1^ in the crab shell leftover samples after 32 h of incubation time. After the fermentation process was completed, the FTIR spectral bands at 3469, 3404, 3606, and 3696 cm^−1^ were attributed mainly correlate to OH– stretching vibration band and were ascribed to intramolecular hydrogen bonds –OH···OC– [55]. Moreover, the O–H stretching vibration and bending hydroxyl groups in Ca(OH)_2_ were shown to cause an absorption peak to appear at 3635 cm^−1^ [56]. The absorption spectra of crab shells leftover samples at zero incubation time demonstrate that the prominent absorption peaks at 2917, 2851, and 2877 cm^–1^ are allocated to asymmetric and symmetric stretching vibrations of the –CH_3_ and –CH_2_ groups [54]. Following 32 h of incubation time, it was clearly shown that the strength of the absorption peak decreased as well as the location of the peak was slightly shifted to 2916, 2850, and 2877 cm^–1^, respectively. Furthermore, the absorption peaks at 1711–1781 cm^−1^, 1739 cm^−1^, and 1740 cm^−1^, which are ascribed to the C = O stretching of carboxylic acids, ester, and aldehyde, respectively, have been identified in the spectrum of crab shells samples. The intermolecular hydrogen bonds between the oxygen atoms of the carbonyl (C=O) group and the hydrogen atoms of the amine (NH_2_) and hydroxyl (OH) groups interconnect the biopolymeric chains, as shown by the FTIR spectrum of the crab shells leftover samples, which showed two distinct peaks of the vibration modes of amide I at 1627 and 1642 cm^−1^ [55]. While, the peak that can be seen at 1584 cm^−1^, in spectra of crab shells leftover samples at zero incubation time, is caused by the amide II bands being bent by the N–H bond. The band attributed to NH_3_^+^ at 1519 cm^−1^ suggests that carboxymethylation proceeded at the OH site, which was shifted to 1506 cm^−1^ after 32 h incubation time of crab shells leftover samples spectra. The characteristic calcite absorption signal was seen at 1435, 470 cm^−1^, which were attributed to CO_3_^−2^ derived from the CaCO_3_ in crab shells leftover sample spectra and dissipated after 32 h of the fermentation process. Moreover, absorption peaks at 1233–1274 cm^−1^ ascribed to group amide III were no longer visible after 32 h of incubation, according to the absorbent spectrum of crab shells [57]. Bridge oxygen stretching was ascribed to the absorption band at 1150 cm^−1^. It was noticed that the deformation of C-H and C-O bonds was indicated by the bands about 1150 and 1132 cm^−1^, which demonstrated the polysaccharide's β-1,4 glycosidic bond, were disappeared after the fermentation process ended [54]. However, once the incubation period ended, the absorption peak at 1028 cm^−1^, which corresponds to a C-O stretching vibration in the spectra of crab shells leftover samples, was relocated to 1017 cm^−1^. In addition, after the fermentation process was completed, the absorption peak at 1195 cm^−1^, which corresponds to the vibration of a C–O–C bond, was displaced to 1188 cm in the spectrum of the crab shells leftover samples. The C–H bonds in the anomeric carbon are responsible for the peaks in the spectra at 897–898 cm^–1^ [46]. Chitin's glucopyranosicyclic residues anomeric centre configuration has been studied using this band as a reference point. In-plane and out-of-plane asymmetric C–O stretching deformation modes may be seen at about 866 and 702 cm^−1^, which correspond to the carbonate group of CaCO_3_. In a sample of crab shells incubated for 32 h, the wagging vibration of the C–H bond may be attributed to the weak absorption peak at 795 cm^–1^, and an absorption band at 657 cm^−1^ was discovered, which was ascribed to the δ (NH) out of plane [58]. Leftover crab shells provided spectra with absorption peaks at 529 and 560 cm^−1^, which corresponded to the bending vibrations of Si–O and O–P–O (*ν4*), respectively. Broad band around 486 and 490 cm^−1^ is caused by the bending vibrations of the hydroxyl and pyranose groups. The peaks at 1268 cm^−1^ and 1229 cm^−1^, which correspond to the acetyl and amide groups, were no longer present in the treated crab shells leftover samples because the bonds between these groups had been broken. Other peaks' intensity changed as well, chitinaceous material has degraded as a result of these alterations [53]. The XRD experiment had a significant role in this debate. Crystal structure orientation and functional properties of organic matrix components in crab shells were evaluated by X-ray diffraction (XRD) analysis of finely ground shells powder leftover samples, which were taken at zero incubation time and after the fermentation process was completed, and the results are shown in a diffractogram as illustrated in Fig. 12b. This data was deemed to be in strong agreement with the Joint Committee on Powder Diffraction Standards (JCPDS). A textural coefficient (*hkl*) was used to determine which direction the preferred orientation should be. The crystal size was calculated using the Scherrer formula: $D={}^{K\lambda }/{}_{\beta \cos \theta }$ where D is the crystallite size, $K$ is a constant of 0.9, $\lambda$ is the *X*-ray wavelength, $\beta$ is full width at half maximum (FWHM), and $\theta$ is the diffraction peak. The diffraction peak with the highest intensity at $\theta$ =29.4 was discovered by XRD investigation. This intensity is characteristic of rhombohedra calcite (CaCO_3_; PDF: 03–0593) and accounts proportion of 26.13% of the total weight of the crab shells leftover samples at zero incubation time. Calcite (CaCO_3_) is the main ingredient of crab shells, according to Boselmann et al. [59], which accounts for a significant portion of the crab shells' overall weight. However, after the fermentation process concluded, the calcium mineralization of the elaborated crab shells was visible in the diffraction intensity of the calcite diffractogram (CaCO_3_; PDF: 03–0593) with a proportion of 31.65%, which shows that the chitin biopolymer matrix is mineralized with tiny crystallites, calcites, and amorphous calcium [60]. Moreover, diffraction patterns were seen with various forms of calcite at $\theta$ values of 23.02, 36.04, 39.1 and 43.04, 47.6, 57.2, 60.9, and 65.2 are identical to those observed with the strongest calcite faces. The high-crystalline calcite (CaCO_3_) was verified in crab shells by Zhou et al. [61], who indexed a collection of diffraction peaks at $\theta$-values of 29.6, 36.2, 39.6, 43.4, 47.7, 48.8 and 57.8. In addition, the XRD patterns of the crab shell leftover samples revealed a significant diffraction peak at $\theta$-value of 19.4, which is indicative of crystal α-chitin. The structural type of chitin generated from crab and shrimp shells has been identified in the literature as α-chitin [62]. The XRD patterns demonstrate that the locations and intensities of the chitin peaks in the crab shells are similar to those of other works from various antecedents in literature. Crab shell powder from Chinese hairy crabs was found to have a diffraction peak at $\theta$-value of 19.4 in its XRD patterns, confirming the presence of crab shell chitin in the samples [61], which was following the current findings. Furthermore, the presence of magnesite (MgCO_3_; PDF: 02-0875) in the crab shells leftover samples was verified by the observation of a prominent peak at $\theta$-value of 46.9 with the lattice plane of (202). At zero incubation time, a substantial peak at $\theta$-value of 30.9 for calcium phosphate oxide [Ca_3_(PO_4_); PDF: 01-0941] with a proportion of 17.61% was seen in the crab shells leftover samples, which was then moved after the fermentation process was complete to a significant peak at $\theta$-value of 31.6 of calcium phosphate [Ca (PO_4_)_2_; PDF: 03-0348] accounts proportion of 12.74%. Apart from the above mentioned, the crab shells included calcium silicate [Ca_2_ SiO_4_; PDF: 01-1029] which was discovered by a diffraction peak at $\theta$-value of 64.4 corresponding to a plane of (360). While, at $\theta$-value of 56.8 with the lattice plane of (535), a faint signal was identified, confirming the presence of silicon oxide [SiO_2_; PDF: 02-0242] in crab shells.

## Conclusions

As shellfish processing produces hazardous pollutants, proper waste management is essential to safeguarding our ecosystem. An enormous reservoir of valuable byproducts exists in the waste products of shellfish processing and they may be financially and ecologically friendly converted utilizing generally recognized as safe (GRAS) microorganisms or regenerated Natural Deep Eutectic solvents, resulting in almost zero waste. The study's main goal was to increase the utilization of crab shells trash and reduce environmental pollution by producing ALP from *B. licheniformis* using an eco-friendly bioprocessing approach. In light of these facts, the most potent ALP-producing *B. licheniformis* strain ALP3 was isolated from tanning and leather industry, sludge effluent, and identified as a local bacterial strain based on genetic and morphological characteristics. A new and innovative nutritive medium formulation with simple and affordable components was designed for the production of ALP using sequential statistical techniques. It was found that the crab shells were shown to be the most significant factor influencing the production processes through PBD, followed by ZnSO_4_.7H_2_O, NaH_2_PO_4_, and CoCl_2_.6H_2_O. One of the most economical ways to boost ALP production efficiency was using bioprocessing scale-up strategies in 7-L bench-top reactors under uncontrolled pH cultivation conditions. It was discovered that the ALP production rate was significantly increased, with a reduction in the production time of approximately 8 h when compared to its shake flask counterpart, when the dissolved oxygen concentration was maintained above 20%. *B. licheniformis* strain ALP3's ALP production was first boosted with PBD (90.06 U L^−1^), then with SCCD (111.6 U L^−1^), and last with uncontrolled pH cultivation (212.29 U L^−1^). Productivity improved by 1.21, 1.51, and 2.87 times, respectively, as compared to the initial media. A significant increase in Co^+^, K^+^, Fe^+^, Cd^2+^, Cr^+^, Zn^+^, Mn^2+^, Ca^2+^, and Na^+^ concentrations was observed after the fermentation process ended, which was a result of the ALP activity on crab shells particles. The findings of a morphological and functional study using TGA; DSC; SEM; EDS; FTIR; and XRD demonstrate that ALP activity has a substantial effect on crab shells particle remediation.

## Material and methods

### Sample collection and isolate sources

The target plant for aseptically collecting sludge samples is Leather & Tanning Factories, Alexandria, Egypt. The samples were relocated to a refrigerator when they arrived at the Bioprocess Development lab and kept at 4 °C until they were used in the following analyses.

### Enrichment and isolation of ALP-producing bacteria

After 48 h incubation time at 45 °C with 200 rpm in an orbital shaker, the ALP-producing bacteria were stimulated following the method reported by Abdelgalil et al. [7], who utilized Pikovskaya (PKV) broth medium, pH 7.0, however, crab shells powder was used as a stimulator for ALP production. A large amount of crab shell waste was collected from seafood restaurants and thoroughly cleansed with distilled water to remove impurities. After that, they were dried for 10 h in a drying oven at 70 °C before being pulverized in a blender and passing through a 0.125-mm sieve. As Abdelgalil et al. [7] showed, the enriched bacteria were isolated, purified, and regularly subcultured on Luria–Bertani (LB) medium agar.

### Qualitative screening for ALP activity

To investigate the isolates' CaCO_3_-solubilizing abilities, PKV's agar enriched with CaCO_3_ was used as a screening medium, while chromogenic substrates such as phenolphthalein diphosphate tetrasodium salt (PDP) and methyl green dye were incorporated into LB agar media as a means of evaluating ALP capability [20]. After inoculating medium agar plates of screening media with the activated pre-cultures, the plates were incubated at 45 °C for an overnight period to screen for the pure isolates. The presence of a clear zone surrounding the colonies on PKV agar plates demonstrated that the isolates possessed the capacity to solubilize calcium carbonate, whereas the emergence of noticeable green district coloured colonies on MG-PDP agar medium indicated that the isolates possessed the ability to produce ALP. To conduct further study on one of the intriguing isolates, ALP3 was picked and subjected to further morphological and molecular identification methods. This isolate was recognized as such because it exhibited the largest solubilization clear zone and the most intense colony colour of all the isolates tested.

### Quantitative screening for ALP activity

For a quantitative evaluation of ALP activity, the crude enzyme was harvested after 48 h of cultivation of one ml of 12 h activated pre-cultures of the selected isolates on 50 ml of PKV broth medium supplemented with crab shells powder (2%) at 45 °C and 200 rpm. It was determined that ALP activity had been achieved in a standard reaction mixture consisting of universal buffer pH 11, *ρ*-nitrophenyl phosphate (NPP), and an enzyme solution in a total volume of 1.1 ml after incubation at 65 °C for 5 min, and the reaction was halted by adding an adequate volume (ml) of 1 M NaOH to the mixture as documented by Hashem et al. [63]. Measurement of the colour change induced by the absorption of *ρ*-nitrophenol at 405 nm (ε = 18,000 M^−1^ cm^−1^) was made using an ultraviolet–visible spectrum spectrophotometer. International units (IU) are used to describe the amount of enzyme that generates 1.0 μmol of *ρ*-nitrophenol in 1 min at pH 11 and 65 °C, and the activities were expressed as U L^−1^ min^−1^.

### Amplification of the *16S rDNA* gene, sequencing, and similarity

The salting-out approach was used to extract the genomic DNA of the most formidable ALP-producing strain [64]. Following that, the methodologies developed by Abdelgalil et al. [65] have been used for the amplification of *16S rRNA* gene fragments, the sequencing of the amplified fragment, and the construction of a neighbour-joining phylogenetic dendrogram to determine evolutionary relations. The phylogenetic analysis of the strain sequences was carried out using the CLUSTAL W method of the MEGA X program. The Kimura correction was used in a paired deletion method to compute distances. For the reconstruction of phylogenetic trees, the MEGA X software package's neighbour-joining (NJ), maximum-likelihood (ML), and maximum-parsimony (MP) algorithms were utilized [66]. A bootstrap approach based on 1000 replications was used to calculate percentage support numbers and to evaluate the internal branches' reliability.

### Morphological investigation of *B. licheniformis* strain ALP3

SEM imaging was carried out at the City of Scientific Research and Technological Applications (SRTA-city) laboratory centres to disclose the cell surface morphology and shape of the most effective ALP-producing bacteria. Using sputtering equipment (JFC-1100 E Joel, USA), a thin coating of gold was sputtered over the dried bacterial thin film to create a gold layer.

### Optimization of the physical parameters for the production of ALP

By using the quasi-optimal protocol outlined by Abdelgalil et al. [7], the effectiveness of ALP production was evaluated for a variety of physical parameters, including temperature, initial pH values, and inoculum size. The pH of 50 ml PKV broth medium supplemented with 2% crab shells powder was individually adjusted to 4.0, 5.0, 6.0, 7.0, and 8.0 using 0.1 M HCl to determine the optimum pH for ALP production. The most efficacious strain's activated pre-culture was utilized for inoculation of the adjusted pH crab shell powdered PKV broth medium, followed by a 48 h incubation in a rotary shaker incubator (200 rpm) at 45 °C before harvesting. Additionally, crab shell powdered PKV broth medium without adjustment of the initial pH value was also investigated. To evaluate the influence of temperature on the ALP production process, inoculated flasks of crab shell powdered PKV broth medium with the strain under investigation were incubated individually for 48 h (200 rpm) in a rotary shaker incubator at four different temperatures (37, 40, 45, and 50 °C). The varied activated inoculum sizes (1–10%) of the chosen strain were employed as pre-inoculums for aerobic cultivation of PKV broth medium supplemented with 2% crab shells powder at 50 °C and 200 rpm to identify the most accurate inoculum size in the ALP production process. Each experiment's cell-free supernatants were used to assess ALP activity.

### Media formulation for ALP production by *B. licheniformis* strain ALP3

It is widely accepted that statistical Design of Experiments (DoE) is the most useful method for scientific information acquisition since it applies to multi-factor interaction studies. Predictive mathematical models have opened up a whole new realm of possibilities for the rational design of the microbial production process [67].

### PBD

A fractional factorial two-level model was used to determine the significant nutrient parameters affecting ALP production and *B. licheniformis* strain ALP3 growth using a 2^k^-PBD (variables, k = 13) with six central points in 26 combination trial batches (twenty main batches plus six central point's batches). Several modifications were made to the original PKV broth constitutes to determine how different nutrimental ingredients influenced ALP production. To determine the most significant factors for the peak production of ALP by identifying *B. licheniformis* in submerged fermentation; the thirteen variables namely; molasses [*X*_*1*_], Arabic gum [*X*_*2*_], ammonium sulfate [*X*_*3*_: NH_4_(SO_4_)_2_], sodium nitrate [*X*_*4*_: NaNO_3_], crab shells powder [*X*_*5*_], sodium chloride [*X*_*6*_: NaCl], magnesium chloride [*X*_*7*_: MgCl_2_·6H_2_O], di-potassium phosphate [*X*_*8*_: K_2_HPO_4_], sodium phosphate [*X*_*9*_: NaH_2_PO_4_], cupper sulphate [*X*_*10*_: CuSO_4_·5H_2_O], zinc sulphate [*X*_*11*_: ZnSO_4_·H_2_O], nickel sulphate [*X*_*12*_: NiSO_4_], and cobalt chloride [*X*_*13*_: CoCl_2_·6H_2_O] were handpicked at random for PBD. ALP activity was chosen as the response with a confidence level of all intervals at 95% to generate regression coefficient values. For each independent variable, there were two levels of investigation: high (+1) and low (−1), which were used to assess the influence of each variable. These levels were used to set the upper and lower bounds of each variable's range, and the six centre points were employed in six different batches for each of the independent variables listed in Table 1. A regression model was constructed using the following equation as a coding scheme for testing variables [68]:

$${x}_{i}=\frac{\left({X}_{i}-{X}_{i}^{*}\right)}{\Delta {X}_{i}}$$

In this case, ${x}_{i}$ is the ith independent variable of the dimensionless coded value; the un-coded value of the ith independent variable is denoted by${X}_{i}$; similarly, the uncoded value of the ith independent variable at the centre point is denoted by ${X}_{i}^{*}$; and the step change value has been defined by$\Delta {X}_{i}$. The main effect was computed as the difference between average measurements of each variable taken at a high level (+1) and a low level (−1) of the variable's variability. Multiple linear regression may be approached in several ways. One approach is to describe the predicted mean response ŷ as a linear combination of the predictors for each experimental run.

$$\hat{y}={\beta }_{0}+\sum_{j=1}^{M}{\beta }_{j}{X}_{ij}$$

where $\hat{y}$ is the predicted response (ALP activity U L^−1^ min^−1^), *β*_*o*_ is the model intercepts, *β*_*j*_ is the linear regression coefficient, and *X*_*ij*_ is the coded independent variables estimates. The concept of hierarchy, which asserts that first-order effects tend to prevail, is a factor in the effectiveness of screening designs despite this severe constraint. To assess the efficiency and feasibility of the regression model, statistical analysis was done on the given experimental data, including ANOVA, coefficient determination, and polynomial model reduction using statistical design free download software packages. All factors were evaluated using the student's *t*-test to determine their significance. For each variable, the *p*-value was calculated to determine whether or not it had a significant impact on the outcome [65]. For each parameter, the Pareto chart was able to demonstrate its orderly standardized influence, while also highlighting each parameter's significance. Statistically significant is the parameter's standardized impact when it exceeds a threshold. It was also determined that the most significant impacts were listed in decreasing significance [7, 20, 65]. The coefficient of multiple determinations (*R*^*2*^) and lack-of-fit value may both be used to assess the model's adequacy. Crab shell powder (*X*_*5*_), ZnSO4·7H2O (*X*_*11*_), and CoCl2·6H2O (*X*_*13*_) were found to have the greatest impact on ALP throughput in this investigation, hence they were selected for further optimization using SCCD. To determine average ALP production at predicted optimum independent variable values and verify those predictions, a confirmation experiment has been performed.

### Response surface methodology (SCCD)

One type of response surface methodology is the Box-Wilson Central Composite Design, which is also known as a central composite design. It is composed of an embedded factorial or fractional factorial design with centre points that are used in conjunction with a number of star points to allow for an estimation of curvature in the design [69]. 2n axial and n_c_ centre points are added to the 2^n^ factorial to form a CCD, which is illustrated in the following equation:$N= {2}^{n}+2n+{C}_{0}$ where N is the actual number of experiments, C_0_ is the number of repetitions of the experiments at the centre point and ‘n’ is the number of independent variables which were incorporated within the study. The axial points (2n), which assess the variance of model prediction and are constant at all sites equidistant from the design centre, are for screening analysis and readability. The use of a spherical CCD for modelling and optimizing was chosen to improve prediction accuracy. The spherical designs are rotatable in that all of the points on the sphere are equally spaced out from the centre point [33]. The response function's variance is referred to as its rotational property. Using a spherical CCD, three independent factors (crab shells powder, *X*_*1*_; ZnSO_4_·7H_2_O, *X*_*2*_; and CoCl_2_·6H_2_O, *X*_*3*_) were correlated with ALP activity (Y) as a dependent response variable, which was selected from PBD as a target to enhance ALP production at five distinct levels labelled as (− α, − 1,0, + 1, + α). With the use of a second-degree polynomial equation, the response was utilized to create an empirical model that was then used to correlate the experimental variables. It is as follows:

$${Y=\beta }_{0}+\sum_{i}{\beta }_{i}{X}_{i}+\sum_{ii}{\beta }_{ii}{X}_{i}^{2}+\sum_{ij}{\beta }_{ij}{X}_{i}{X}_{j}$$

where *Y* is the predicted response (ALP activity U L^−1^ min^−1^); *β*_*0*_ is the model intercept; *X*_*i*_ and *X*_*j*_ are the independent variables, *β*_*i*_ is linear coefficients; *β*_*ij*_ is the cross-product coefficients; *β*_*ii*_ is the quadratic coefficients. A fixed distance α (in this case, 1.73205) from the centre generated the quadratic terms, from which 2n axial points were established and the repetitions were performed to prevent error; where n is the number of independent variables. A precise description of curvature and interactions makes this second-order polynomial model appropriate for optimization and inference of nontrivial processes. It was determined that optimizing the three medium factors that had the greatest impact on ALP output and made the most substantial contribution could be accomplished by creating 40 experiments of 2^k^-factor spherical central composite design with 16 cube points, 12 centre points, and 12 axial points (Table 3). Fitting the models represented by a quadratic equation, as well as evaluating the statistical significance of the model terms, were accomplished via the use of regression analysis and analysis of variance (ANOVA) approaches. ANOVA is a mathematical procedure for partitioning the variability of a bunch of data into assignable causes and establishing different significance tests. The statistical performance of the developed model was evaluated using a variety of statistical criteria, including the *F*-value, the *p*-value, *t*-test, the coefficient of determination (*R*^*2*^), the adjusted *R*^*2*^, the predicted *R*^*2*^, the coefficient of variation (C.V.), the lack of fit, and the Predicted Residual Sum of Squares (PRESS) [67]. Three-dimensional surface plots of the fitted equations, which were constructed using statistical software, were used for visualization of each factor's response and experimental levels as well as for determining optimal conditions [7, 20, 65]. We were able to construct and assess statistical experimental designs with the assistance of a statistical designs software that was available for download. Statistics were used to verify that ALP production took place as predicted under shake flask conditions, allowing for an accurate evaluation of the equation model and the exact computations of theoretical values for each variable. The average findings of each experiment, executed in triplicate and independently, are shown in Table 3.

### Bioprocess strategies for scale-up production of bacterial ALP

In the present study, the purpose was to investigate the growth kinetics of *B. licheniformis* strain ALP3 in a submerged cultivation system by increasing the ALP production process from shake flask to benchtop bioreactor scale.

### Shake-flask batch cultivation

Batch mode shake flask experiments were carried out for 32 h of the cultivation period using 250-ml Erlenmeyer flasks, each with 50 ml of the sterile optimized ALP production medium [(g L^−1^); crab shells powder, 46.3; NaNO_3_, 0.3; K_2_HPO_4_, 0.06; ZnSO_4_·7H_2_O, 0.0042; CoCl_2_·6H_2_O, 0.0046; without pH adjustment] at 50 ± 2 °C and 200 rpm. A 12-h-old inoculum was utilized to inoculate a 4% (v/v) production medium. Fermentation samples were taken at intervals of two h to measure biomass as well as ALP activity, total carbohydrates, a total soluble phosphate, and total soluble protein concentrations over the course of the fermentation process. For evaluating the reproducibility of the findings, three batches of experiments were conducted.

### Stirred-bioreactor batch cultivation system

The studies were conducted in a 7.0 L (4-L working volume) stirred tank bioreactor (Bioflow 310, New Brunswick Scientific, USA) to verify the optimal fermentation medium for ALP production at a large scale as described by Abdelgalil et al. [20]. In this study, all of the stirred tank bioreactor cultivations were conducted under the same cultural conditions (inoculum size, temperature, and pH) as the shake flask cultivation system. The stirrer was outfitted with two six-bladed Rushton turbine impellers for maximum efficiency. To maintain dissolved oxygen concentrations above 20%, it was established that the agitation speed should be between 200 and 350 rpm, and this was followed throughout the cultivation process. Filtered sterile air was continuously supplied at a rate of 0.5 v/v/min for aeration, preventing the formation of foam was accomplished by adding an antifoam agent (0.5% silicon oil, 10% vinegar). Changes in pH and dissolved oxygen were tracked throughout the cultivation process using pH and DO polarographic electrodes (Ingold, Mittler-Toledo, Switzerland). However, under a controlled pH cultivation condition, a pH controller in combination with acid/base feeding peristaltic pumps connected to 2 M HCl and 2 M NaOH solutions kept the pH constant at 8.7. On the computer screen, the culture parameters (DO, pH, temperature, and agitation) were displayed, and they were saved to a hard drive for further analysis and interpretation. In pre-weighed, sterile falcon tubes of 50 ml, a total of 20 ml of culture samples were collected at various intervals throughout the 32 h fermentation process. Using a Beckman DU spectrophotometer, the light absorbance at 600 nm of collected samples was compared to a blank to track cell growth. Centrifugation of the withdrawn cultural samples at 10,000 rpm for 15 min was used to collect the cell-free supernatant, which was then utilized for further analytical investigation, while the obtained cell pellets were used to determine the dry cell biomass, as described by Abdelgalil et al. [7].

### Analytical procedures

#### Determination of total carbohydrates

Morris et al.'s [70] anthrone sulfuric acid method was used to quantitatively estimate the total carbohydrate content in the culture filtrate.

#### Determination of total soluble phosphate and protein content

The soluble phosphate content and protein content of the bacterial culture filtrate were determined using the ascorbate and Lowry methods, respectively [71, 72].

### Morphological structure and energy-dispersive spectroscopy (EDS) analysis of the eggshells

At the SRTA-city laboratory centre, a scanning electron microscope (Jeol jsm 6360 LA, Japan) coupled with an in-situ EDS spectrophotometer was used to study the surface morphology of crab shells leftover samples as well as the energy-dispersive X-ray before and after submerged cultivation of bacterial cells.

### Particle size analysis

Before and after the fermentation process of *B. licheniformis* strain ALP3, the SRTA-city laboratory centre employed a particle size analyzer (PSA; Mod.: N5, Beckman Coulter, city, state abbreviation, USA) to measure the particle size of the leftovers of crab shells.

### Atomic absorptions analysis

Samples of crab shells leftover from the cultivation process were evaluated for heavy metal concentrations using AAS (Zeenit 700 Analytik Jena, Germany) at SRTA-laboratory City's centre.

### Thermal analysis (TGA–DSC)

Thermogravimetric analyzer (TGA, Model 50/50H, Shimadzu, Japan), and a differential scan calorimeter (DSC) were used at the Faculty of Science-Alexandria university laboratory centre to evaluate crab shell residual samples' thermal properties and pyrolysis patterns. A nitrogen atmosphere (flow rate of 20 ml min^−1^) was used for TGA and DSC analysis, which was carried out at a constant heating rate of 10 °C min^−1^. On the graph, the relationship between temperature and weight loss was shown (percentage).

### Fourier-transform infrared spectroscopy (FT-IR)

The Shimadzu FTIR-8400 S, Japan, has been used at the SRTA-city laboratory centre to evaluate the active chemical bonds or functional groups associated with crab shells leftover samples before and after the cultivation process. In this experiment, the KBr disc approach was used as the matrix, scanners were used to scan a mid-IR spectrum with a resolution of 4 cm^−1^ in the range of 4000 to 400 cm^−1^.

### X-ray diffraction (XRD) analysis

The crystal structure, phase, and texture of crab shells leftover samples were identified using an X-ray diffractometer (Bruker MeaSrv [D2-208219]) with CuK (k = 1.54A°) radiation and scanned within a 2° to 100° 2θ angular range at a scan speed of 0.02°/s at the faculty of science laboratory centre, Alexandria university.

## Data Availability

All data produced during this study are included in this published article.

## Abbreviations

ALP: Alkaline Phosphatase
AAS: Atomic Absorption Spectrometry
ANOVA: Analysis of Variance
AT%: Atomic percentage
*B*: *Bacillus*
CCD: Central Composite Design
CV: Coefficient of Variation
DF: Desirability Function
DSC: Differential Scan Calorimeter
EDS: Energy-Dispersive Spectroscopy
FTIR: Fourier-Transform Infrared Spectroscopy
JCPDS: Joint Committee on Powder Diffraction Standards
h: Hour
LB: Luria–Bertani
NPP: *ρ*-Nitrophenyl Phosphate
PBD: Plackett–Burman Design
PDP: Phenolphthalein Diphosphate tetrasodium salt
PKV: Pikovskaya
PRESS: Predicted Residual Sum of Squares
TGA: Thermogravimetric Analyzer
RSM: Response Surface Methodology
SCCD: Spherical Central Composite Design
STRs: Stirred-Tank Reactors
XRD: X-Ray Diffraction
